# A Multiomics, Molecular Atlas of Breast Cancer Survivors

**DOI:** 10.3390/metabo14070396

**Published:** 2024-07-20

**Authors:** Brent A. Bauer, Caleb M. Schmidt, Kathryn J. Ruddy, Janet E. Olson, Cem Meydan, Julian C. Schmidt, Sheena Y. Smith, Fergus J. Couch, John C. Earls, Nathan D. Price, Joel T. Dudley, Christopher E. Mason, Bodi Zhang, Stephen M. Phipps, Michael A. Schmidt

**Affiliations:** 1 Mayo Clinic, Rochester, MN 55905, USA; bauer.brent@mayo.edu (B.A.B.);; 2 Sovaris Aerospace, Boulder, CO 80302, USA; 3 Advanced Pattern Analysis and Human Performance Group, Boulder, CO 80302, USA; 4 Thorne Research, Inc., Summerville, SC 29483, USA; 5 Buck Institute for Research on Aging, Novato, CA 94945, USA

**Keywords:** breast cancer survivors, breast cancer, multiomics, genomics, metagenomics, metabolomics, proteomics, microbiome, omega-3 fatty acids, aptamer

## Abstract

Breast cancer imposes a significant burden globally. While the survival rate is steadily improving, much remains to be elucidated. This observational, single time point, multiomic study utilizing genomics, proteomics, targeted and untargeted metabolomics, and metagenomics in a breast cancer survivor (BCS) and age-matched healthy control cohort (N = 100) provides deep molecular phenotyping of breast cancer survivors. In this study, the BCS cohort had significantly higher polygenic risk scores for breast cancer than the control group. Carnitine and hexanoyl carnitine were significantly different. Several bile acid and fatty acid metabolites were significantly dissimilar, most notably the Omega-3 Index (O3I) (significantly lower in BCS). Proteomic and metagenomic analyses identified group and pathway differences, which warrant further investigation. The database built from this study contributes a wealth of data on breast cancer survivorship where there has been a paucity, affording the ability to identify patterns and novel insights that can drive new hypotheses and inform future research. Expansion of this database in the treatment-naïve, newly diagnosed, controlling for treatment confounders, and through the disease progression, can be leveraged to profile and contextualize breast cancer and breast cancer survivorship, potentially leading to the development of new strategies to combat this disease and improve the quality of life for its victims.

## 1. Introduction

Breast cancer (BrCa) remains a formidable global health challenge, affecting more than two million women and their families each year and leading to almost 700,000 deaths globally [1]. In 2020, female breast cancer had the highest incidence rate of all cancers in both sexes and in female-specific cancers, accounting for 11.7% of all cancers and 24.5% of female cancers [1]. In the United States, breast cancer accounted for an estimated 31% of new female cancer cases and an estimated 15% of female cancer-related deaths in 2022 [2]. As such, women with breast cancer represent the largest sub-population of those with cancer in the United States and worldwide.

There exists a body of literature on molecular analytics and therapeutics in breast cancer [3,4,5,6,7,8,9,10,11,12,13,14,15,16,17], including an emergent body of literature with multiomics applied to biopsy specimens in breast cancer [4,6,9,10,11,14,15,17]. However, there is a paucity of research in which multiomic analyses have been applied to breast cancer survivors (BCS) who have completed treatment. 

The present study was established as part of a two-step investigation to address this need. The first step, reported herein, was designed to apply a comprehensive multiomic analysis (using untargeted genomics, proteomics (serum), metabolomics (plasma, urine, stool), gut metagenomics, and polygenic risk scores) to a cohort of BCS derived from the Mayo Clinic Breast Cancer Registry. This includes the most detailed NextGen sequencing of the gut metagenome in BCS to date.

The primary aim was to develop a comprehensive molecular atlas of a single time point comparing a BCS cohort with an age-matched control group of individuals with no history of breast cancer. The resultant molecular atlas would form the foundation upon which future multiomics investigations into a larger BCS cohort might be based.

The second step was envisioned to refine the paradigm and apply those lessons to a multiomic study of those newly diagnosed with breast cancer and prior to treatment initiation. An acknowledged limitation of the present study is the confounding effect of treatment on the molecular dynamics in BCS, wherein it is not possible to draw conclusions about how the molecular findings may or may not have contributed to the evolution of the disease. However, the present study does lend insight into whether the molecular phenotypes in the BCS vs. the control cohorts were convergent or divergent. In general, the application of the methods reported in this paper is expected to, in the future, provide the ability to describe the molecular dynamics in those newly diagnosed with breast cancer in greater detail than has been reported previously. From this study, and future studies building upon this platform, new insights and hypotheses can emerge which strengthen, deepen, and potentially improve the way breast cancer is approached, before, during, and after diagnosis.

## 2. Materials and Methods

### 2.1. Study Design, Recruitment, Inclusion/Exclusion Criteria, and Informed Consent

In this study, we performed advanced multiscale omics analysis coupled with gut microbiome metagenome and gut microbiome metabolome data analyses in Breast Cancer Survivors (BCS). This included univariate, multivariate, and pathway analysis applied to large datasets developed to detect unique patterns of variance in targeted serum metabolites, plasma metabolites, gut microbiome community structure, gut microbiome metabolome, urine metabolome, and quality of life measures. These measures have aided in the development of a database that can be used as a reference population for precision medicine in BCS.

Cases and controls were recruited to the study via mail or in person. Each study packet included an informed consent document, an invitation letter, and a return envelope. For BCS cases, eligible participants were adult females enrolled in the Mayo Clinic Biospecimen Resource for Breast Disease (IRB # 1815-04), current ages 18–75 years, with a prior diagnosis of stage 0–3 (in situ or invasive) breast cancer (BCS) who had completed active therapy (surgery, radiation, and/or chemotherapy) approximately 8–30 months prior to provision of their samples. For trial controls, age-matched (within 0–5 years depending upon the convenience and availability) females with no history of cancer (other than non-melanoma skin cancer) were selected from the PRISM Study (IRB #18-002366 The Predicting Risk after Screening Mammogram (PRISM) Study) or the Mayo Clinic Breast Mammography practice. Exclusion criteria for both cohorts (BCS cases and trial controls) exclusion criteria included: male biological sex or gender, pregnant females, or participants unwilling to travel to Mayo Clinic Rochester for the study visit or unwilling to provide mail-in stool samples.

Participants came to the Mayo Clinic Clinical Research and Trials Unit (Mayo Clinic CRTU) in Rochester, MN, USA. At the CRTU, participants were asked to sign the consent form, their height and weights were then obtained, questionnaires were completed, and the participants’ samples were obtained. Some data was gathered via electronic health record (EHR) or was from other studies in which these individuals had participated (Mayo Clinic Biospecimen Resource for Breast Disease (IRB # 1815-04) or IRB #18-002366: The Predicting Risk after Screening Mammogram (PRISM Study)). Both avenues of additional data collection were consented to by participants prior to enrollment in this study.

Descriptive statistics (mean; median; SD; IQR) were obtained for BCS and HC for parametric variables. For PHQ-8 and GAD-7, the mean and standard deviation were analyzed. For significance testing of parametric variables, Student’s *t*-tests were conducted. For non-parametric statistics, the Wilcoxon Rank-Sum Test was performed. All statistical analysis for metadata was performed using R version 4.2.3.

### 2.2. Whole Genome Sequencing

The WGS protocol used in this experiment was similar to the protocol previously described by Meydan et al. and will be briefly summarized here [18]. Stool samples were collected using Thorne’s Gut Health Test, which provides metagenomic sequencing of the microbiome. To isolate microbial DNA from the samples, an automated protocol, and MoBio’s PowerMag^®^ (+CleaMag^®^) microbiome DNA isolation kit were utilized on the KingFischer^TM^ Flex Instrument. The concentration of extracted DNA from each sample and estimated sample purity were determined by Qubit measurement and spectrophotometry (A260/280 and A260/230 absorbance ratios), respectively.

Nextera XT Library Prep (Illumina, San Diego, CA, USA) was used to enzymatically fragment and tag primer sites for adapter index addition, creating next-generation sequencing libraries. Sequencing adapters and indices were added during polymerase chain reaction (PCR) amplification, followed by library verification by fragment analysis (Agilent Bioanalyzer, Agilent, Santa Clara, CA, USA). DNA sequencing was performed utilizing the Illumina NextSeq platform, which generated 350 M reads per sample (150 × 150 read length) and translated to 24× coverage.

Whole genome sequences were aligned to the hg38 reference genome using BWA mem with the default parameters [19]. Sentieon LocusCollector and Dedup algorithms were used to remove duplicate reads [20]. After indel realignment and base recalibration using Sentieon default parameters, Somatic SNVs, and SVs were called with the DNAscope algorithm [21]. Known variants were annotated using dbSNP SNVs, Indels, and Mills, and 1000 genomes indels [22,23]. After variant calling, variants were filtered by QC, including removing any sites with *p* > 0.25 or marked as low quality.

Polygenic risk score (PRS) matrices were collected from PGS Catalog [24] for signatures of breast cancer-specific scores and other breast cancer subtypes, as well as genetic signatures for 54 health and disease scores [25]. PRS for each individual was calculated by scoring the variants of the subject using the PGS Catalog calculator (PGS Catalog Calculator (in preparation [0]). PGS Catalog Team. PGScatalog/pgsc_calc). Statistical comparison of healthy controls versus breast cancer survivors was performed on these scores using the Wilcoxon rank-sum test.

### 2.3. Proteomics via Aptamer

Proteomic profiling was conducted at SomaLogic Operating Co., Inc. (Boulder, CO, USA), using the SomaScan Assay. The SomaScan method has been previously described in detail by Gold and colleagues and Kim et al. [26,27]. Briefly, SOMAmer (Slow Off-rate Modified Aptamers) reagents consist of short single-stranded DNA sequences with a high binding affinity for proteins. The diverse chemical properties allow for a greatly expanded library from which SOMAmer reagents are selected. SOMAmer reagents are discovered in vitro using the SELEX (Systemic Evolution of Ligands by EXponential enrichment) process, which incorporates chemically modified nucleotides with structural similarity to desired amino acid side chains. This enhances the specificity and affinity of the target protein binding interaction.

Initially, samples were diluted and incubated with SOMAmer reagent mixes attached to streptavidin (SA)-coated beads. The beads had been washed and tagged with an NHS-biotin reagent. SOMAmer complexes and unbound SOMAmer reagents were subjected to ultraviolet light to cleave a “photo-cleavable” linker within the SOMAmer reagent which released them into a solution containing an anionic competitor. The anionic competitor displaced the SOMAmer reagents in the eluent, which was then incubated with a second SA-coated bead. Subsequent washing removed the free SOMAmer regent, followed by a final elution step, in which the protein-bound SOMAmer reagent was released from the proteins when mixed with a denaturing agent. Quantification of SOMAmer reagents was achieved by hybridization to custom DNA microarrays, which were detected via their cyanine-3 signal.

Principle component analysis was performed to reduce the dimensionality of a dataset by identifying and retaining the most significant features. The distance matrix from generalized unifrac metrics was fed into t-distributed stochastic neighbor embedding (t-SNE) to project the samples into a two-dimensional space [28]. Log-normal univariate (Model A) and multivariate modeling (Model B and Model C) were performed. Model A was an uncorrected comparison for BCS versus healthy cohort. Model B corrected for age and menopause status and Model C corrected for age, menopause, and treatment type. Pathway enrichment analysis was performed, and the top 50 pathways were presented for each model.

### 2.4. Targeted Metabolomics

#### 2.4.1. Acylcarnitines

The following acylcarnitines were analyzed in human plasma samples: carnitine, 2-methyl butyryl carnitine, 3-hydroxy butyryl carnitine, acetylcarnitine, butyryl carnitine, decanoyl carnitine, hexanoyl carnitine, isobutyryl carnitine, isovaleryl carnitine, lauroyl carnitine, linoleoyl carnitine, myristoyl carnitine, octanoyl carnitine, oleoyl carnitine, palmitoyl carnitine, propionyl carnitine, stearoyl carnitine, and valeryl carnitine. Samples were analyzed via LC-MS/MS by Metabolon, Inc. (Morrisville, NC, USA). The plasma samples were spiked with stable, labeled internal standards, homogenized, and subjected to protein precipitation with organic solvents. Following centrifugation, an aliquot of the supernatant was injected into an Agilent 1290/AB Sciex QTrap 5500 LC-MS/MS system equipped with a C18 reversed-phase UHPLC column. The mass spectrometer was operated in positive mode using electrospray ionization (ESI).

The peak of the individual analyte product ions was measured against the peak of the corresponding internal standards. Quantitation was performed using a weighted linear least squares regression analysis generated from fortified calibration standards and prepared immediately prior to each run.

LC-MS/MS raw data were collected and processed using AB SCIEX software Analyst 1.6.3 and SCIEX OS-MQ software v1.7. Data reduction was performed using Microsoft Excel for Office 365 v.16.

#### 2.4.2. Bile Acids

A Human Plasma Bile Acid Panel was performed, which measures all the primary and secondary bile acids and their conjugates. Bile acid concentrations were analyzed by LC-MS/MS (Metabolon Method TAM178: “LC-MS/MS Method for the Quantitation of Bile Acids”). Bile acids measured included: cholic acid (CA), chenodeoxycholic acid (CDCA), deoxycholic acid (DCA), lithocholic acid (LCA), ursodeoxycholic acid (UDCA), glycocholic acid (GCA), glycochenodeoxycholic acid (GCDCA), glycodeoxycholic acid (GDCA), glycoursodeoxycholic acid (GUDCA), taurocholic acid (TCA), taurochenodeoxycholic acid (TCDCA), taurodeoxycholic acid (TDCA), taurolithocholic acid (TLCA), tauroursodeoxycholic acid (TUDCA), and lycolithocholic acid (GLCA).

Calibration samples were prepared at eight different concentration levels by spiking a phosphate-buffered saline/bovine serum albumin (PBS/BSA) solution with a corresponding calibration spiking solution. Calibration samples, study samples, and quality control samples were spiked with a solution of isotopically labeled internal standards and subjected to protein separation with acidified methanol (organic solvent). Following centrifugation, an aliquot of supernatant was evaporated and dried in a stream of nitrogen. The dried extracts were reconstituted and injected on an Agilent 1290 Infinity/SCIEX QTRAP 6500 LC-MS/MS system equipped with a C18 reverse phase UHPLC column. The mass spectrometer was operated in negative mode using electrospray ionization (ESI).

The peak area of each bile acid parent (pseudo-MRM mode) or the product ion was measured against the peak area of the respective internal standard parent (pseudo-MRM mode) or the product ion. Quantitation was performed using a weighted linear least squares regression analysis generated from fortified calibration standards prepared immediately prior to each run.

LC-MS/MS raw data were collected using SCIEX software Analyst 1.7.3 and processed using SCIEX OS-MQ v.1.7. Data reduction was performed using Microsoft Excel for Office 365 v.16.

#### 2.4.3. Fatty Acid Panel

In order to assess red blood cell (RBC) fatty acids, dried blood spot (DBS) samples were collected according to manufacturer instructions (OmegaQuant Analytics, LLC.; Sioux Falls, SD, USA). Samples were processed and analyzed according to OmegaQuant Omega-3 Index^®^ methodology, as follows. A drop of blood was collected on filter paper that was pre-treated with a cocktail solution (Fatty Acid Preservative Solution, FAPS™) and allowed to dry at room temperature for 15 min. Following drying, samples were kept at 2–8 °C. The DBS was shipped to OmegaQuant for fatty acid analysis. One punch of the DBS was transferred to a screw-cap glass vial followed by the addition of BTM (methanol containing 14% boron trifluoride, toluene, methanol; 35:30:35 *v*/*v*/*v*; Sigma-Aldrich, St. Louis, MO, USA). The vial was briefly vortexed and heated in a hot bath at 100 °C for 45 min. After cooling, hexane (EMD Chemicals, Gibbstown, NJ, USA) and HPLC-grade water were added. Subsequently, the tubes were recapped, vortexed, and centrifuged to help separate layers. An aliquot of the hexane layer was transferred to a gas chromatography (GC) vial. GC was carried out using a GC-2010 Gas Chromatograph (Shimadzu Corporation, Columbia, MD, USA) equipped with an SP-2560, 100-m fused silica capillary column (0.25 mm internal diameter, 0.2 um film thickness; Supelco, Bellefonte, PA, USA).

Fatty acids were identified by comparison with a standard mixture of fatty acids characteristic of RBC (GLC OQ-A, NuCheck Prep, Elysian, MN, USA) which was also used to construct individual fatty acid calibration curves. The following 24 fatty acids (by class) were identified: saturated (14:0, 16:0, 18:0, 20:0, 22:0 24:0); cis monounsaturated (16:1, 18:1, 20:1, 24:1); trans (16:1, 18:1*, 18:2*—see below for more details); cis n-6 polyunsaturated (18:2, 18:3, 20:2, 20:3, 20:4, 22:4, 22:5); cis n-3 polyunsaturated (18:3, 20:5, 22:5, 22:6). Fatty acid composition was expressed as a percent of total identified fatty acids. The Omega-3 Index is defined as the sum of 20:5n-3 (EPA) and 22:6n-3 (DHA) adjusted by a regression equation (r = 0.97) to predict the Omega-3 Index in the RBC.

* The chromatographic conditions used in this study were sufficient to isolate the C16:1trans isomers and the C18:2 D 9t-12c, 9t-12t, and 9c-12t isomers; the latter is reported as C18:2n6t. However, each individual C18:1 trans molecular species (i.e., C18:1 D6 through D13) could not be separated but appeared as two blended peaks that eluted just before oleic acid. The areas of these two peaks were summed and referred to as a C18:1 trans.

#### 2.4.4. Targeted Metabolomics Statistical Methods

Due to non-normal distributions, acylcarnitine, bile acid, and RBC fatty acid data were subjected to Mann Whitney U Tests (non-parametric, between groups) and Kruskal-Wallis ANOVA (non-parametric, select findings) (OriginPro 2024 (64-bit) SR1 ver 10.1.0.178). Data were Log_10_ normalized and presented as split violin plots to enhance visualization.

### 2.5. Untargeted Metabolomics

Untargeted metabolomics analyses were conducted on stool, plasma, and urine samples. Stool samples were homogenized by sonification after adding 1 × PBS (10 mL per mg tissue) prior to prepping. Plasma, urine, and stool homogenates were deproteinized with 6× volume of cold acetonitrile:methanol (1:1) solution following the addition of 13C6-phenylalanine (3 µL at 250 ng/)µL as internal standard. Samples were kept on ice with intermittent vortexing and centrifuged at 18,000× *g* for 30 min at 4 °C. The supernatants were divided into 2 aliquots and dried down for analysis on a Quadruple Time-of-Flight Mass Spectrometer (Agilent Technologies 6550 Q-TOF, San Diego, CA, USA) coupled with an Ultra High-Pressure Liquid Chromatograph (1290 Infinity UHPLC Agilent Technologies, San Diego, CA, USA). Profiling data were acquired under both positive and negative electrospray ionization conditions over a mass range of 100–1200 *m*/*z* at a resolution of 10,000–35,000 (separate runs). Metabolite separation was achieved using two columns of differing polarity, a hydrophilic interaction column (HILIC, ethylene-bridged hybrid 2.1 × 150 mm, 1.7 mm; Waters) and a reversed-phase C18 column (high-strength silica 2.1 × 150 mm, 1.8 mm; Waters). For each column, the run time is 20 min using a flow rate of 400/µL min. A total of four runs per sample was performed to give maximum coverage of metabolites. A quality control sample, made up of a subset of samples from the study, was injected several times during each run. All raw data files obtained were converted to compound exchange file format using Masshunter DA reprocessor software (Agilent, San Diego, CA, USA). Mass Profiler Professional (Agilent, San Diego, CA, USA) was used for data alignment and to convert each metabolite feature (*m*/*z* × intensity × time) into a matrix of detected peaks for compound identification. Putative Identifications (IDs) were given to components based on mass molecular weights and further examined by comparison to a reference standard of the proposed compound. The mass accuracy of the Q-TOF method was <5 ppm with retention time precision better than 0.2%. A 1.2× fold change can be detected with a precision of 4%. 13C6 phenylalanine internal standard was used to check for recovery of each sample only and not in normalization of the data.

An unsupervised principal component analysis, ANOVA, 3D plot and heat map, and a Partial Least Square discrimination analysis (PLS-DA) comparison between groups were generated for analysis. The R-package MetaboanalystR software (version 3.3.0) was used for data normalization, differential expression analysis, and visualization. In each mode, metabolites were row-wise normalized to a constant sum (SumNorm), log-transformed, and then scaled by mean centering (MeanCenter). Normalized data were analyzed by multivariate Principal Component Analysis (PCA) to reveal data heterogeneity, groupings, outliers, and trends. Hierarchical clustering analysis (HCA) was performed to reveal clustering between sample injections, group replicates, and multiple metabolite clusters that correlate with different subsets of clinical variables. The univariate Student’s unpaired *t*-test was conducted for between-group analysis with multiple testing corrections to identify differentially expressed metabolites between two groups with statistical significance (FDR adjusted *p*-value ≤ 0.05 and |fold change| ≥ 1.5).

### 2.6. Gut Metagenomics

Gut metagenomics analyses were performed at CosmosID (Germantown, MD, USA). DNA from stool samples was isolated using the QIAGEN DNeasy PowerSoil Pro Kit, according to the manufacturer’s protocol. DNA samples were quantified using the GloMax Plate Reader System (Promega, Madison, WI, USA) using the QuantiFluor^®^ dsDNA System (Promega, Madison, WI, USA) chemistry. DNA libraries were prepared using the Nextera XT DNA Library Preparation Kit (Illumina, San Diego, CA, USA) and IDT Unique Dual Indexes with a total DNA input of 1 ng. Genomic DNA was fragmented using a proportional amount of Illumina Nextera XT fragmentation enzyme. Unique dual indexes were added to each sample followed by 12 cycles of PCR to construct libraries. DNA libraries were purified using AMpure magnetic beads (Beckman Coulter) and eluted in QIAGEN EB buffer. DNA libraries were quantified using Qubit 4 fluorometer and Qubit™ dsDNA HS Assay Kit. Libraries were then sequenced on an Illumina NextSeq 2000 platform 2 × 150b.

Sequences were trimmed by Trimmomatic [29], and then aligned to human genome reference using BWA [19]. Taxonomic annotation was performed by utilizing KrakenUniq [30] and subsequently Bracken [31] on a database that includes all bacterial, archaeal, viral, and fungal references from RefSeq along with human references. The lowest common ancestor taxonomic annotations were adjusted within the lineage until at least 10% of the unique k-mers belonged to a specific clade and not its parent, then filtered for at least 10 reads and a minimum Bracken-adjusted relative abundance of 0.005%. Pearson correlation was calculated by taking the log abundances of the species (or other relevant ranks) and comparing these between the two samples. The functional annotations for genes were performed by using HUMAnN3 with the UniRef90 clusters and summarized as MetaCyc v19.1 pathways by HUMAnN3 [32]. The alpha diversity analysis was performed using Shannon entropy and species richness on the Bracken results of each replicate. Beta diversity was calculated as a generalized unifrac distance between the samples [33,34]. Statistical analyses of beta diversity were performed by comparing the within-group distance to inter-group distances. Differential abundance was calculated using MaAsLin2 with a negative binomial model and trimmed mean of M-values normalization (TMM) [35,36]. Differentially abundant taxa were modeled in three ways: (1) BCS vs. HC, (2) BCS grouped by stage at diagnosis, and (3) BCS grouped by cancer subtype. The correction factors were age, as well as the age and menopause state at the time of sampling. For differentially abundant taxa, taxon disease annotation was accomplished via the Disbiome database [37].

### 2.7. Quality of Life Questionnaires

Participants each electronically completed the Generalized Anxiety Disorder-7 questionnaire (GAD-7) and the Patient Health Questionnaire-8 (PHQ-8). Results were used as a means to assess the differences in the general quality of life (QoL) between the two groups. The PHQ-8 consists of 8 questions (0–3 points each) focused on anxiety (4 questions) and depression (4 questions). Scores range from 0–24 and are interpreted as 5–9 mild, 10–14 moderate, 15–19 moderately severe, and 20+ severe depression. The GAD-7 consists of 7 questions (0–3 points each) focused on generalized anxiety. Scores range from 0–21 and are interpreted as 5–9 mild, 10–14 moderate, and 15+ severe anxiety. Wilcoxon rank-sum tests were performed on the total score from each questionnaire.

## 3. Results

**Figure 1:**
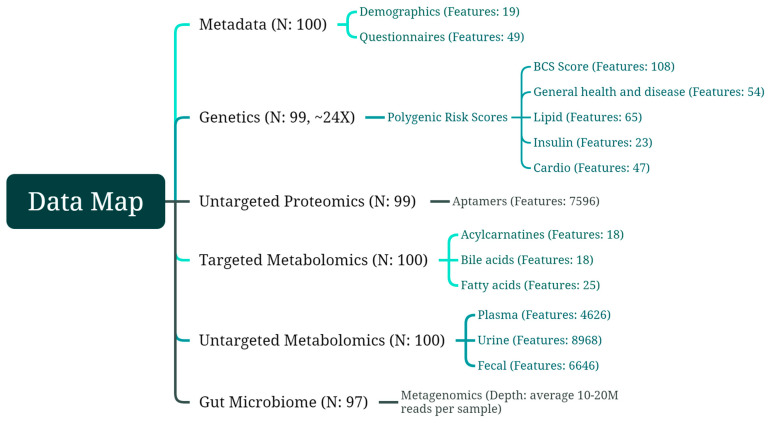
Data map showing classes of data with a number of subjects, detailed metrics assessed within each class, and a number of features measured within each metric.

To accomplish the stated objectives of this single time point, observational controlled study, metadata were gathered by questionnaire, taken (blinded for the study team) from electronic health records of BCS participants, and analyzed from biological samples (blood, urine, feces). Whole genome sequencing (WGS) from blood yielded polygenic risk scores via analysis of single nucleotide polymorphisms (SNP). Targeted metabolomics panels included acylcarnitines and bile acids from plasma and fatty acids from RBCs. Untargeted proteomics was analyzed from plasma and assessed via aptamers. Untargeted metabolomics assays were run on plasma, urine, and stool to unmask unknown or unexpected differences. Metagenomics via NextGen sequencing was completed for the microbiome from stool samples. The data map in Figure 1 outlines these multiomic features and the following sections report the results from each class of data gathered.

### 3.1. Metadata: Description of the Cohort

**Figure A1:**
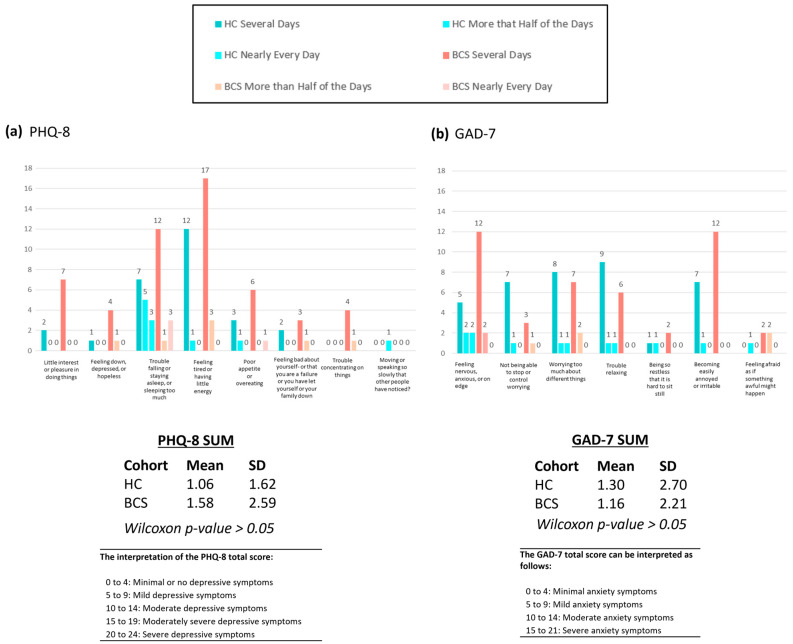
Questionnaire Responses for (**a**) PHQ-8 and (**b**) GAD-7.

Data were collected from a total of 100 female participants comprised of two equal groups—50 Breast Cancer Survivors (BCS) and 50 healthy controls (HC). These results are shown in Table 1. The mean age of all participants was 63 years (BCS = 62.8; HC = 63.2). Participants assigned to the BCS group had a prior diagnosis of stage 0–3 (in situ or invasive) breast cancer and had completed active therapy (e.g., chemotherapy, radiotherapy, endocrine therapy, or some mixture of treatment modalities), with a mean-time from treatment of 518 days (IQR: 411–613 days) prior to provision of the samples. Questionnaire data for the PHQ-8 and the GAD-7 are shown in Figure A1a,b, respectively. There were no significant findings between the two cohorts, in regards to the responses to those questionnaires. 

### 3.2. Genetic Analysis via Whole Genome Sequencing

**Figure 2:**
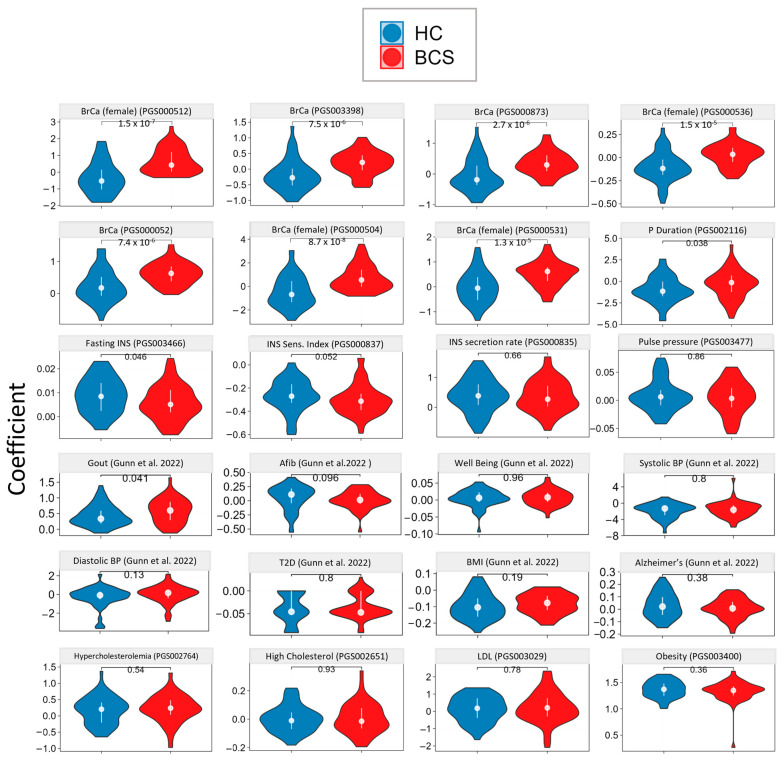
Selected Polygenic Risk Scores. These scores emphasize the significant predisposition for breast cancer risk and the relative homogeneity of the population in regard to other PRSs assessed. BrCA: breast cancer, INS: insulin, BP: blood pressure, BMI: body mass index, LDL: low-density lipoprotein [38].

Whole genome sequencing (WGS) was completed for each participant in both cohorts. Using these data, we applied multiple polygenic risk score (PRS) calculations to the cohort in order to understand their genetic predisposition for health and disease. PRS categories included breast cancer (108 scores), lipid (65 scores), insulin (23 scores), cardio (47 scores), and general health and disease (54 scores) [38]. These were assessed for a total of 297 individual scores. The complete results for BrCa-specific PRSs are reported in Appendix B (Table A1), with selected PRSs for BrCA and other disease signatures shown in Figure 2.

As a first-pass genetics analysis, we expected this would give us the best overarching look at genetic differences between the HC and BCS cohorts. Within this analysis, two major themes stood out: (1) the genetic homogeneity present between these two age-matched cohorts and (2) the significant predisposition for BrCa risk present in the BCS cohort. Of the 189 non-breast cancer-specific PRSs assessed, significant differences were found in only 7. Two *p*-wave duration scores (*p* = 0.043 and 0.038) and gout (*p* = 0.041) were significantly higher in BCS vs control, while fasting insulin (*p* = 0.046) and insulin sensitivity index (*p* = 0.052), were significantly lower in BCS compared to HC. In the BrCa risk PRS category, 73 of the 108 PRSs assessed showed significance (q < 0.05), indicating a strong genetic predisposition for a genetic component of BrCa. This battery of predispositions correlated well with BrCa incidence in the BCS cohort (as the BCS cohort was defined by participants who had dealt with BrCa and recovered successfully).

### 3.3. Targeted Metabolomics

The investigation of targeted metabolomics was restricted to three important molecular classes with relevance to BCS and their largely yet-to-be-observed dynamics in each of the following: acylcarnitines (AC), bile acids (BA), and omega fatty acids (OFA). More specifically, ACs were investigated due to their relevance to dysfunctions in energy metabolism, BA was investigated due to their relevance to other types of cancers, and fatty acids were investigated because of their relevance in inflammatory balance.

#### 3.3.1. Acylcarnitines

**Figure 3:**
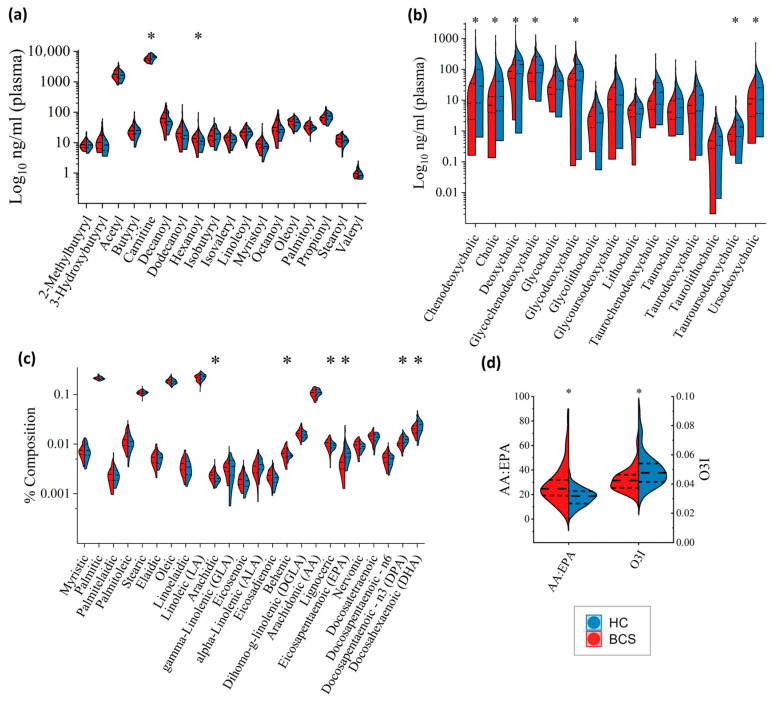
Targeted Metabolomics. Split violin plots showing BCS on the left (red) and HC on the right (blue) with individual analytes of each type plotted on a Log10 scale. (**a**) Acylcarnitines (ng/mL); (**b**) Bile Acids (ng/mL); (**c**) Fatty Acids (% composition); (**d**) AA:EPA Ratio (arachidonic acid:eicosapentaenoic acid) and Omega-3 Index. * Wilcoxon Rank-Sum Test between-groups *p*-value < 0.05.

**Figure A2:**
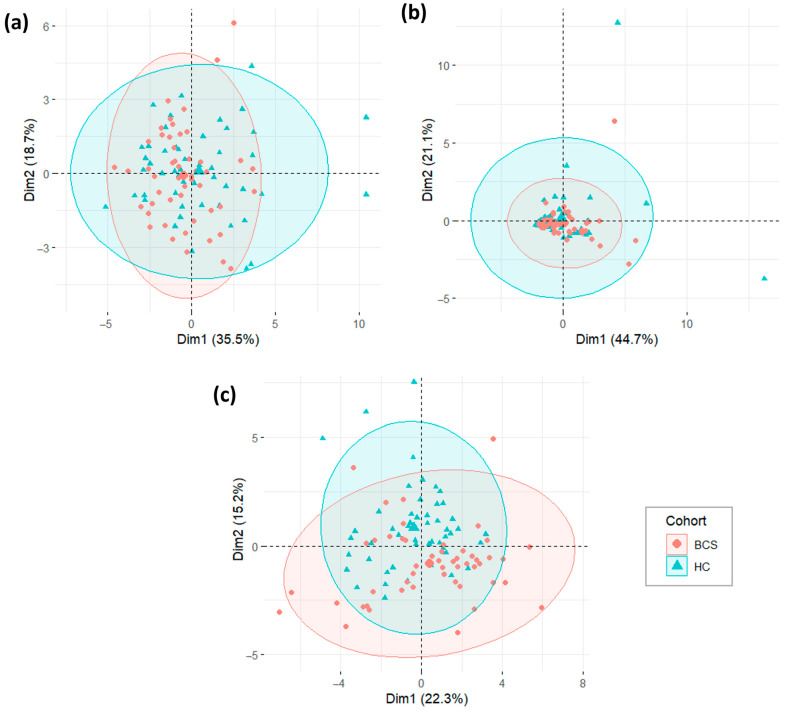
Targeted Metabolomics. (**a**) Acylcarnitines PCA, (**b**) Bile Acid PCA, (**c**) O3 FA PCA.

Acylcarnitines were analyzed in plasma samples using LC-MS/MS. Eighteen acylcarnitines were selected for evaluation including carnitine, 2-methyl butyryl carnitine, 3-hydroxy butyryl carnitine, acetylcarnitine, butyryl carnitine, decanoyl carnitine, hexanoyl carnitine, isobutyryl carnitine, isovaleryl carnitine, lauroyl carnitine, linoleoyl carnitine, myristoyl carnitine, octanoyl carnitine, oleoyl carnitine, palmitoyl carnitine, propionyl carnitine, stearoyl carnitine, and valeryl carnitine. Overall, principal component analysis (PCA) plots show a high degree of similarity between the BCS and HC cohorts (Figure A2a). Of the 18 acylcarnitine metabolites, only carnitine (*p* = 0.0358; lower) and hexanoyl carnitine (*p* = 0.0410; higher) were found to be significantly different between BCS and healthy controls (Figure 3a).

#### 3.3.2. Bile Acids

Bile acids were analyzed in plasma samples using LC-MS/MS. As was the case with acylcarnitines, PCA plots for bile acids showed similarities between the BCS and HC cohorts (Figure A2b). However, nearly half of them differed, with 7 of the 15 analyzed bile acids significantly altered in BCS: Chenodeoxycholic acid (CDCA; *p* = 0.0023), tauroursodeoxycholic acid (TUDCA; *p* = 0.0109), cholic acid (CA; *p* = 0.0245), glycodeoxycholic acid (GDCA; *p* = 0.0249), glycochenodeoxycholic acid (GCDCA; *p* = 0.0257), and two secondary bile acids deoxycholic acid (DCA; *p* = 0.0361), and ursodeoxycholic acid (UDCA; *p* = 0.0396), all significantly lower in BCS compared to healthy controls (Figure 3b).

#### 3.3.3. RBC Fatty Acids

Twenty-four fatty acids were analyzed in red blood cells using gas chromatography (GC). PCA plot analysis showed a high degree of similarity between the two groups (Figure A2c). Six of the 24 analytes were significantly altered in BCS compared to HC (Figure 3c). Eicosapentaenoic Acid (EPA; *p* = 4.16 × 10^−5^), Docosahexaenoic Acid (DHA; *p* = 0.0071), and Docosapentaenoic Acid (DPA (n − 3); *p* = 0.007), all omega-3 polyunsaturated fatty acids, were significantly lower in BCS compared to controls, whereas arachidic acid (*p* = 0.0027), lignoceric acid (*p* = 0.031), and behenic acid (*p* = 0.0498) were significantly higher in BCS compared to controls (Figure 3c). Additionally, the Omega-3 Index (EPA + DHA; *p* = 4.92 × 10^−4^) was significantly lower in BCS than in healthy controls (Figure 3d). Arachidonic acid (AA) levels were not significantly different. However the AA:EPA (arachidonic acid:eicosapentaenoic acid ratio) ratio was significantly higher (*p* = 1.0 × 10^−5^) in BCS with a value of 28.07 compared to 18.00 in healthy controls (Figure 3d).

### 3.4. Aptamer-Based Untargeted Proteomics

#### 3.4.1. Primary and Exploratory Proteomic Analysis

**Figure 4:**
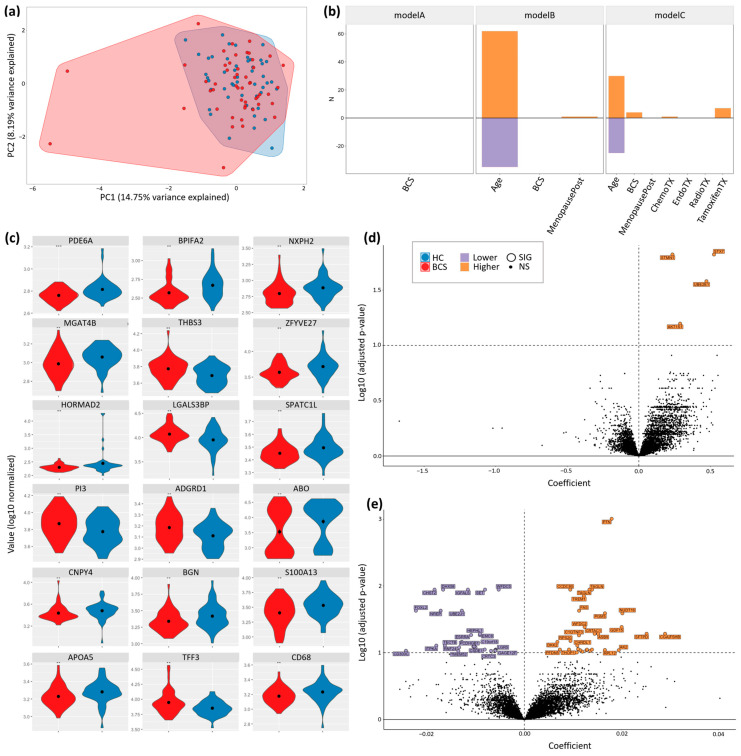
Untargeted Proteomics. (**a**) PCA of BCS Serum Proteomics (lognormal filtered, by type, SD = 0.5); (**b**) Univariate and Multivariate Models (q < 0.1): Model A—BCS Status; Model B—BCS status, age, and menopause status; Model C—BCS status, age, menopause status, and treatment type; (**c**) BCS vs. HC: significantly different proteins (after multiple hypothesis correction; log10 normalized); (**d**) Volcano plot of Model C by BCS status; (**e**) Volcano plot of Model C by age; ** and *** Wilcoxon Rank-Sum Test *p*-value < 0.01 and *p*-value < 0.001, respectively. Note: Gene names are used for brevity, however, all findings in this figure refer to gene products (proteins).

**Figure A3:**
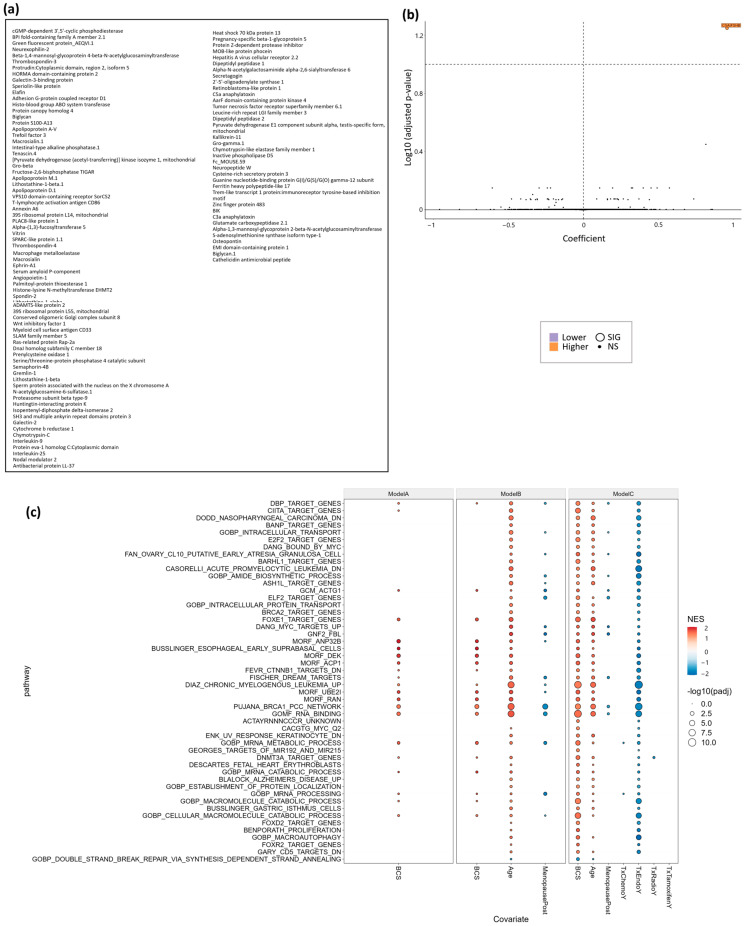
Additional Untargeted Aptamer Proteomics. (**a**) 108 exploratory proteomics targets, (**b**) multivariate Model B for age, (**c**) pathway analysis of proteomics data.

PCA analysis revealed a significant overlap between the two cohorts with several outliers (Figure 4a). PC1 represented 14.75% of the variance, while PC2 represented 8.19% of the variance. The initial exploratory untargeted proteomic analysis showed 108 proteins that were significantly different between BCS and HC (Figure A3a).

Multiple hypothesis corrections narrowed this list to 18 proteins that differed significantly between the two cohorts (Figure 4c). These proteins are: cGMP-dependent 3′5′-Cyclic Phosphodiesterase (PDE6A, *p* = 8.75 × 10^−4^), BPI Fold-containing Family A Member 2.1 (BPIFA2, *p* = 0.00103), Neurexophilin-2 (NXPH2, *p* = 0.002099), Thrombospondin-3 (THBS3, *p* = 0.002937), Beta-1,4-Mannosyl-Glycoprotein 4-Beta-N-Acetylglucosaminyltransferase (MGAT4B), *p* = 0.002305), Protrudin:Cytoplasmic Domain, Region 2, Isoform 5 (ZFYVE27, *p* = 0.00344), HORMA Domain-containing Protein (HORMAD2, *p* = 0.004394), Galectin-3-Binding Protein (LGALS3BP, *p* = 0.00454), Speriolin-like Protein (SPATC1L, *p* = 0.00588), Elafin (PI3, *p* = 0.006145), Adhesion G-protein Coupled Receptor D1 (ADGRD1, *p* = 0.006832), Histo-Blood Group ABO System Transferase (ABO, *p* = 0.007125), Protein Canopy Homolog 4 (CNPY4, *p* = 0.008074), Biglycan (BGN, *p* = 0.0084147), Protein S100-A13 (S100A13, *p* = 0.0084149), Apolipoprotein A-V (APOA5, *p* = 0.008768), Trefoil Factor 3 (TFF3, *p* = 0.008676), and Macrosialin 1 (CD68, *p* = 0.009711).

#### 3.4.2. Univariate and Multivariate Proteomic Analysis

We ran several log-normal univariate and multivariate models on the proteomic data. The first model (Model A) found no features that significantly differentiate between BCS and HC (Figure 3b). The second model (Model B), contrasting BCS and HC while correcting for age and menopause status, found more than 60 proteomic features that were higher and 30 proteomic features that were lower as a function of age (Figure 4b). In this same model, BCS cohort status and menopause status (except for FSH) showed no significant differentiation (Figure 4b and Figure A3b).

In a third model (Model C) of BCS versus HC, which corrected for age, menopause, as well as treatment type, age was again the largest differentiator of significant proteomic features, with more than 30 proteins being higher and 30 proteins being lower (Figure 4e). BCS cohort status and treatment type showed several higher proteomic features in this model (Figure 4b). Additionally, Model C found that stathmin (STMN1), syntaxin-7 (STX7), and the ubiquitin-conjugating enzyme E2 E1 (UBE2E1) were lower in the BCS cohort (Figure 4d).

Figure A3c shows the enrichment of biological pathways based on the proteomic analysis in the context of Models A, B, and C used for the analysis above. Pathways of interest included the BRACA1 PCC network, FoxE1 target genes, chronic myelogenous leukemia up, and GOMF RNA binding domains, among others, which were significantly enriched (up/down) in all models (Figure A3c).

### 3.5. Untargeted Metabolomics

#### 3.5.1. Untargeted Plasma Metabolomics

**Figure 5:**
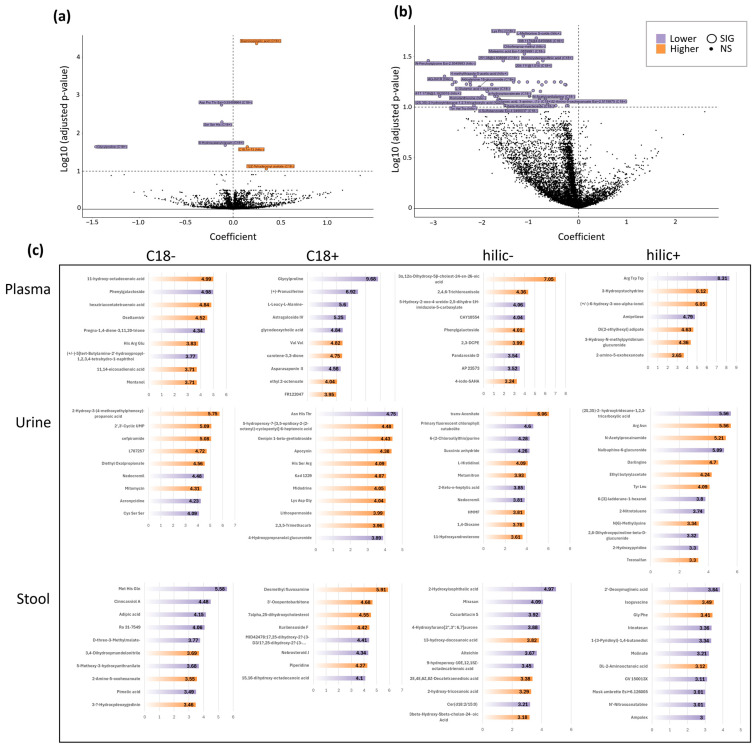
Untargeted Metabolomics. (**a**) Model B of BCS in plasma, (**b**) Model C of ChemoTX in urine, (**c**) VIP scores for enriched metabolites by mass spectrometry model for three sample types (plasma, urine, stool).

**Figure A4:**
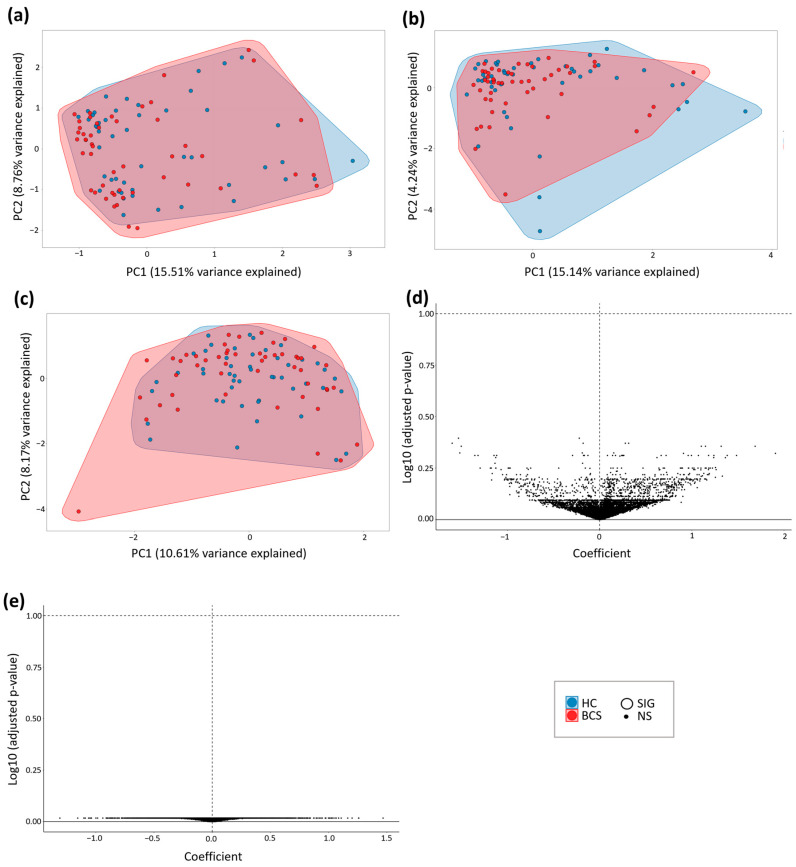
Additional Untargeted Metabolomics. (**a**) PCA of untargeted plasma metabolomics, (**b**) PCA of untargeted urine metabolomics, (**c**) PCA of untargeted stool metabolomics, (**d**) Model B volcano plot of untargeted urine metabolomics, (**e**) Model B volcano plot of untargeted stool metabolomics.

PCA yielded a high overlap between BCS and HC cohorts, indicating a similarity between the two groups (Figure A4a), in regards to urine and stool metabolomics, respectively. Variable importance plots (VIP) for different MS modes highlighted major metabolites of interest (Figure 5c). In multivariate modeling, when controlling for breast cancer type, age, and menopause status in the BCS versus HC comparison, 3 metabolites were higher and 4 metabolites were lower in the BCS cohort (Figure 5a). The molecules that were significantly higher included diaminopimelic acid (*p* = 2.646 × 10^−5^), 12Z-tetradecyl acetate (*p* = 0.0422), and one metabolite that could not be annotated. The metabolites that were significantly lower in BCS compared to HC were Asp-Pro-Thr (*p* = 0.0045), Ser-Ser-His (*p* = 0.0045), glycylproline (*p* = 0.0075), and 8-hydroxyalanylclavam (*p* = 0.0451; Figure 5a).

#### 3.5.2. Untargeted Urine & Stool Metabolomics

PCA yielded a high overlap between BCS and HC cohorts, indicating a similarity between the two groups (Figure A4b,c). When controlling for breast cancer type, age, menopause, and treatment type (tamoxifen, chemo, endo, radio) using multivariate modeling (Model C), 48 identifiable metabolites (shown in Figure 5b and listed in Table A2) were found to be significantly lower (q < 0.1). When controlling for breast cancer type, age, and menopause status (Model B), no significantly different metabolites were found to be higher or lower in either the untargeted urine or untargeted stool results (Figure A4d,e).

#### 3.5.3. VIP Plots and Metabolite Type Annotation

For each of the three sample types (plasma, serum, and stool), a PLS-DA comparison revealed that 122 analytes were of importance. Of those metabolites, 63 were found to be higher in the BCS cohort and 59 were found to be lower (Figure 5c). An analysis aimed at deeper annotation of those 122 metabolites represented in the VIP plots showed that 82 (67%) of the metabolites had some biological or chemical function that was annotated in the literature. The remaining 41 (33%) metabolites had no easily identifiable function. These 82 metabolites were grouped into four categories: (1) known drug (N: 28, 34%), (2) of dietary or supplement origin (N: 17, 21%), (3) endogenous metabolite (N: 14, 17%), or 4) exogenous metabolites or known toxicant (N: 23, 28%). When comparing the BCS cohort to the healthy control, of the: (1) drug metabolites 14 were higher and 14 were lower, (2) dietary or supplement metabolites, 11 were higher and 6 were lower, (3) endogenous metabolites, 7 were higher and 7 were lower, and (4) exogenous or known toxicant metabolites, 9 were higher and 14 were lower.

### 3.6. Gut Microbiome Metagenomics

#### Stool Microbiome

**Figure 6:**
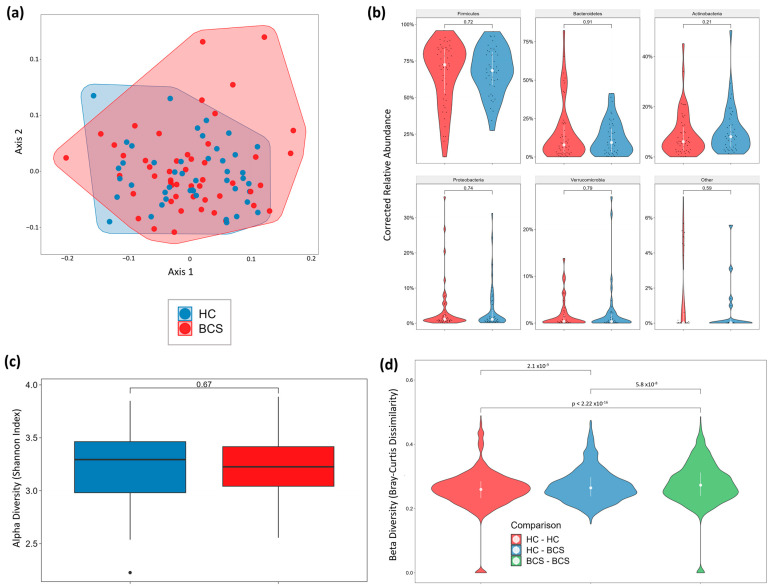
Gut Microbiome Metagenomics I. (**a**) PCoA, (**b**) Phylum Distribution, (**c**) Alpha diversity (Shannon index), (**d**) Beta Diversity (Bray-Curtis Dissimilarity).

**Figure 7:**
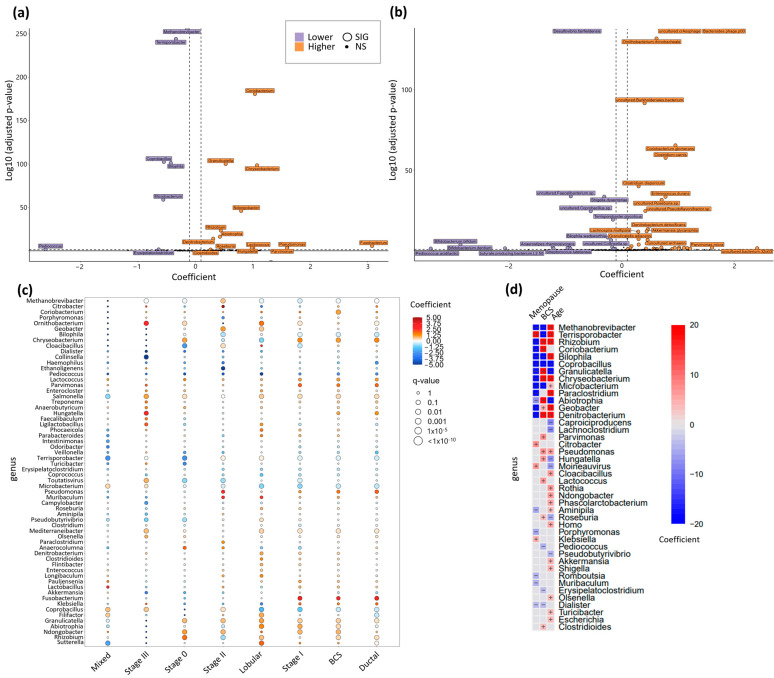
Gut Microbiome Metagenomics II. (**a**) genus volcano plot of BCS vs. HC; (**b**) species volcano plot of BCS vs. HC; (**c**) significant genus grouped by stage at diagnosis and BrCa subtype; (**d**) genus corrected for menopause and age.

**Figure A5:**
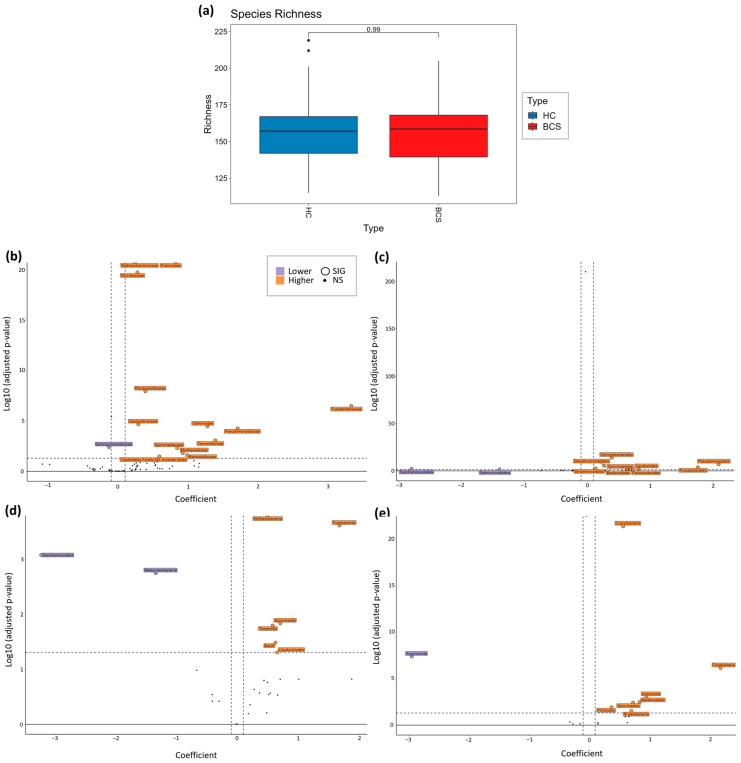
Additional Gut Microbiome Volcano Plots. (**a**) Species Richness between cohorts, (**b**) Volcano Plot by Microbial Family, (**c**) Volcano Plot by Microbial Order, (**d**) Volcano Plot by Microbial Class, (**e**) Volcano Plot by Microbial Phylum.

In the dataset, where the majority of samples fell within the ~10–20 million range, PCA and Principal Coordinates Analysis (PCoA) highlighted a noticeable overlap of cohorts with minor separation (Figure 6a). No significant global differences were observed in phylum distribution (Figure 6b), alpha diversity (Shannon index; Figure 6c), or species richness (Figure A5a). However, beta diversity (Bray-Curtis dissimilarity) analysis revealed several noteworthy distinctions, indicating unique community structure profiles between the two cohorts (Figure 6d). The volcano plots for genus and species revealed separation between the two cohorts in a large number of features (Figure 7a,b). Similarly, the volcano plots for family, order, class, and phylum revealed separation in a number of features that varied between plots (Figure A5b–e).

**Figure 8:**
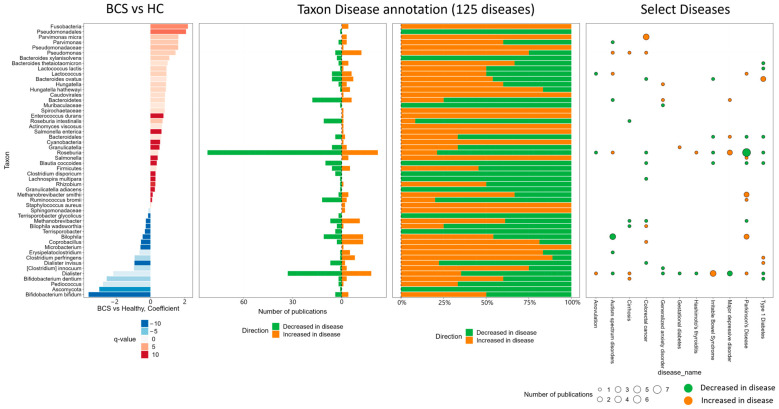
Gut Microbiome Metagenomics III. BCS v. HC taxon disease annotations and selected disease similarities.

Utilizing univariate and multivariate models to account for factors such as stage at diagnosis, breast cancer type, age, and menopause status uncovered substantial differences in the abundance of specific genera and species (Figure 7a–d). Importantly, these distinctions remained consistent across models. Furthermore, delving into the published literature (via a proprietary database) unveiled associations between the identified genus and species differences and a diverse range of diseases with microbiome correlates (Figure 8).

## 4. Discussion

### 4.1. Cohort

This age-matched, entirely female, primarily post-menopausal cohort was statistically similar across groups in all measured demographic variables except anxiety, which occurred more frequently in the BCS cohort. Participants provided samples from 93 to 1043 days post-treatment end. The BrCa types and stages as well as modes of treatment were heterogeneous in this study, potentially confounding the identification of patterns but important in terms of the broader application of this data set as a foundational database to build upon for future work.

### 4.2. Genetics

Specific to this study, we found a difference in preexisting risk between the two cohorts, which emphasizes the usefulness of polygenic scores as a screening measure in women to assess their risk of BrCa. Of the 189 non-breast cancer-specific PRSs assessed, significant differences were found in only 7 scores (3.2%). This finding was evidence of the homogeneity of the cohort (for issues beyond BrCa) and success in age-matching participants. Alternatively, the BCS cohort had a significantly higher predisposition for breast cancer when compared to the healthy controls (73 of 108 scores significantly different between groups or 67.6%). BrCa is known to be a disease where genetics are predictive of future risk. Studies have found that combined risk from single nucleotide polymorphisms (SNPs) is associated with breast cancer in Genome-Wide Association Studies (GWAS) and explains over 30% of breast cancer heritability [39].

### 4.3. Untargeted Proteomics

Univariate modeling (Model A) comparing only BCS status yielded no significant differences between the groups. When correcting for age and menopause in multivariate models in BCS vs. healthy controls (Model B), follicle-stimulating hormone (FSH) was significantly higher in BCS (Figure A3b). Interestingly, a 2010 study found a positive association between serum FSH concentrations and better prognosis during tamoxifen therapy in a cohort of postmenopausal breast cancer patients [40]. Another group of researchers found that Her-2+ patients had higher serum FSH levels compared to Her-2- patients and patients with high Ki67 expression also had higher levels of FSH [41].

With the multivariate Model C, we corrected for age, menopause, and treatment and found that Stathmin (STMN1), Syntaxin-7 (STX7), and Ubiquitin-conjugating enzyme E2 E1 (UBE2E1) were higher in the BCS cohort. STMN1 has been associated with breast cancer due to its influence on cell proliferation, differentiation, and motility. Phosphorylation of the four serine residues of STMN1 leads to inhibition of microtubule polymerization [42]. Furthermore, phosphorylated STMN1 contributes to the regulation of cell migration, cell invasion, and cancer metastasis. Specifically, phosphorylation at serine residues 25 (ser25) and 38 (ser38) of STMN1 was shown to be increased in cells with higher metastatic potential and was also associated with increased morbidity and mortality in breast cancer patients. Following phosphorylation at ser25 and ser38, STMN1 binds to glucose-regulated protein of molecular mass 78 (GRP78). GRP78 is an endoplasmic reticulum chaperone and heat shock protein that is known to be involved in tumor proliferation, survival, and metastasis. In contrast to phosphorylation at ser25 and ser38, phosphorylation at ser16 and ser63 of STMN1 led to improved morbidity and mortality. Researchers also noted that inhibition of MEK kinase, which specifically phosphorylates ser25 and ser38, strongly reduced GRP78 binding. This led the researchers to believe that tumor progression was impacted by site-specific phosphorylation of STMN1 at ser25 and ser38 and subsequent GRP78 binding [43]. In breast cancer tissue, one study found stathmin expression was associated with tumor proliferation, p53 status, basal-like differentiation, BRCA1 genotype, high-grade histology, tumor angiogenesis, immune response, and survival. Essentially, linking stathmin expression to increased severity and worse outcomes in breast cancer [44]. Another study found that STMN1 can be used as a prognostic indicator, based on elevated STMN1 leading to a worse prognosis in breast cancer patients [45].

STX7 is a member of the SNARE family of proteins, which are generally associated with vesicle trafficking. Specifically, STX7 has been associated with the fusion of late endosomes with lysosomes and the homotypic fusion of lysosomes as well as the fusion of lysosomes with phagosomes [46]. Although little research exists on the role of STX7 in breast cancer, a recent in vitro study highlighted the involvement of STX7 as a promoter of invadopedia (i.e., matrix-degrading structures) formation during cancer cell invasion. Thus, increased levels of STX7 are implicated as a major contributing factor in breast cancer cell invasion [47].

Ubiquitin-conjugating enzymes (E2s) are involved in breast cancer progression and are hypothesized to increase resistance in triple-negative breast cancer [48]. There are a number of E2 enzymes that can contribute to breast cancer pathology, playing a myriad of roles depending on the specific enzyme. Elevated UBE2E1 is an interesting finding in BCS. Previously, UBE2E1 has been implicated in acute myelogenous leukemia (AML) and pancreatic cancer. A study of UBE2E1 in AML found that patients with high UBE2E1 expression were non-responsive to chemotherapy and had worse prognoses while those with lower UBE2E1 were more responsive and likely to enter complete remission [49]. UBE2E2 has been shown to promote cancer cell movement and invasion in breast cancer cells through its action on ISG15 [50]. For a complete review of ubiquitin-conjugating enzymes and their involvement in breast cancer, as well as many other cancers, see the works of Du et al. [51] and Voutsadakis [52].

### 4.4. Targeted Metabolomics

#### 4.4.1. Acylcarnitines

Dysregulation in energy metabolism and associated obesity are well-established risk factors for BrCa, especially in postmenopausal women [53]. Further, there is a known link between metabolic disorders and aberrant acylcarnitine metabolism [54], which was the impetus for assessing acylcarnitine in this study. Our results indicated that hexanoyl carnitine was significantly higher and carnitine was significantly lower in the BCS cohort compared to healthy controls. To our knowledge, this is the first study to analyze acylcarnitines in a cohort of breast cancer survivors. However, previous studies have assessed acylcarnitine levels in breast cancer patients. A study published in 2013 by Shen et al. found that hexanoyl carnitine levels were significantly higher in breast cancer patients compared to controls [55]. The majority of our results suggest that BCS patients become more like their healthy counterparts as time goes on. In this instance, however, the significantly elevated hexanoyl carnitine levels from our study of BCS are consistent with the significantly elevated hexanoyl carnitine levels of breast cancer patients found by Shen. Another study found that serum carnitine levels were significantly lower in breast cancer patients compared to controls [56]. This finding by Ozmen et al. shares similarities with our finding of significantly decreased carnitine. However, the Ozmen group measured serum samples and we analyzed plasma samples. Additionally, their group only analyzed patients who had undergone radiotherapy. Therefore, only a general, and not a direct, comparison can be made between the breast cancer patients of the Ozmen study and the BCS of our study.

#### 4.4.2. Bile Acids

The role of bile acids in breast cancer (and cancer, in general) is complex and warrants further investigation and clarification, as much is still unknown. Furthermore, contradictory results make interpretation of the role of bile acids in breast cancer even more difficult. Although assessments of bile acids in BCS vs. healthy controls have not been previously conducted to our knowledge, bile acids have been studied in breast cancer as well as many other cancer types. Our results showed that chenodeoxycholic acid (CDCA), tauroursodeoxycholic acid (TUDCA), cholic acid (CA), glycodeoxycholic acid (GDCA), glycochenodeoxycholic acid (GCDCA), deoxycholic acid (DCA), and ursodeoxycholic acid (UDCA) were significantly lower in BCS patients compared to healthy controls.

CDCA, a major primary bile acid, generally induces oxidative stress, DNA damage, and inflammation. CDCA has been implicated as a tumor promotor in several cancers, however, it has been suggested that CDCA is a tumor suppressor in breast cancer [57]. One study showed that CDCA inhibits the proliferation of cells in tamoxifen-resistant breast cancers via FXR activation [58]. However, another team of researchers reported that the activation of FXR is positively correlated with estrogen receptor expression and supports cell proliferation [59]. Another study comparing the bile acid profiles in the serum of breast cancer patients to patients with benign breast disorder (BBD) found that CDCA was elevated in breast cancer patients [60].

A study by Costarelli and Sanders found that the mean concentration of DCA was 52% higher in breast cancer patients compared to controls, leading them to suggest that DCA may be involved in the etiology of breast cancer, potentially as a tumor promoter [61]. A recent cell study showed that Clostridium-specific DCA plays a molecule-specific role in breast cancer cell proliferation. The researchers found that DCA significantly promoted the proliferation of HER2+ cells but did not affect triple-negative breast cancer cells [62].

Interestingly, UDCA, TUDCA, and GUDCA have been studied as potential treatments for other types of cancer but not specifically for breast cancer. A study analyzing breast tissue samples from breast cancer patients found that several bile acids, including DCA and GCDCA, accumulate in breast tumors. This study also found that the accumulation of these bile acids showed an inverse relationship with proliferation scores. Increased bile acids led to decreased tumor cell proliferation, which led to improved prognostic outcomes [63].

#### 4.4.3. RBC Fatty Acids

In the current study, targeted analysis of RBC fatty acids in BCS patients revealed significantly increased levels of arachidic acid, lignoceric acid, behenic acid, and Omega-3 Index as well as significantly lower levels of EPA, DHA, and DPA. Additionally, the AA:EPA ratio was significantly higher in BCS patients. Significantly altered omega-3 fatty acids may be a contributing factor in the etiology and progression of breast cancer. Furthermore, depleted omega-3 fatty acids may increase susceptibility to the development of breast cancer, impact the effectiveness of treatment, and play a role in survivorship. As such, fatty acid supplementation may be an effective adjunct therapeutic intervention in breast cancer.

Previous studies have found changes in omega-3 fatty acid status in breast cancer patients. For example, Cala et al. found significant changes in 10 fatty acids in their breast cancer cohort of Colombian Hispanic women [64]. Pakiet et al. assessed the serum fatty acid profiles of breast cancer survivors to examine if fatty acid levels normalized following successful treatment. They found a lack of normalization of fatty acid profiles after breast cancer resection, as well as significant differences in serum fatty acid levels before and at 12- and 24-month follow-ups. Interestingly, they found significantly increased levels of EPA and DPA relative to control factors [65]. Shen et al. also found a significant increase in DPA in patients with triple-negative breast tumors compared to the control factors [55]. DHA, behenic acid, and arachidic acid were found to be significantly increased in breast cancer patients with invasive ductal carcinoma [66].

Hidaka et al. looked at the relationship between fatty acid levels and cytologic atypia, a condition that often leads to breast cancer. Their research team found that EPA and DHA were significantly lower in women with cytologic atypia in multiple blood lipid compartments. Additionally, they found that the EPA+DHA:AA ratio, the omega-3:6 ratio, and DPA were all significantly lower in plasma triacylglycerides of women with atypia [67].

A recent review by Fabian et al. presented studies showing a high intake ratio of marine omega-3 fatty acids EPA and DHA relative to the omega-6 fatty acid arachidonic acid reduces the risk of breast cancer compared to those with low intake ratios [68]. Similarly, other studies have shown that a higher dietary intake ratio of omega-3:omega-6 is associated with a lower risk of breast cancer [69,70].

While more research is needed to fully elucidate the role of omega-3 fatty acids in breast cancer and breast cancer survivorship, this study adds to a growing body of evidence implicating omega-3 fatty acids as an important contributing factor in breast cancer progression. Based on our results, we believe it is pertinent for all breast cancer patients to have their omega-3 fatty acid status assessed. Furthermore, we believe that omega-3 fatty acids have potential as an adjunct therapeutic intervention for breast cancer patients.

### 4.5. Untargeted Metabolomics

Our findings from the untargeted plasma metabolomics analysis showed several three-amino acid sequences that were significantly decreased in breast cancer patients compared to healthy controls (i.e., Asp-Pro-Thr and Ser-Ser-His). Recently, an interesting review highlighted research findings that specific amino acids are altered by 10–15% in breast cancers [71]. Lai et al. showed that alanine (Ala), histidine (His), threonine (Thr), arginine (Arg), proline (Pro), glutamate (Glu), and glycine (Gly) are six times more likely to be decreased than increased in cancer patients [72]. An interesting meta-analysis of metabolomics in the diagnosis of breast cancer highlighted the frequency of specific amino acids mentioned in the literature. They reviewed diagnosis-related studies that noted either increased or decreased amino acids in various tissue samples (saliva, blood, urine, and breast tissue). Seven studies found changes in His (3 up and 4 down). However, only one of the seven studies showed a decrease in His in plasma. Six studies found changes in Pro (3 up and 3 down), six studies found changes in Ser (5 up and 1 down), five studies found changes in Thr (2 up and 3 down), and four studies found changes in Asp (3 up and 1 down) [73]. Jobard et al. found that in premenopausal women Ser was associated with a decreased breast cancer risk [74]. As for Asp, results show that it both increases [75] and decreases [76] with the risk of breast cancer. Studies have shown that increased levels of both His and Thr increase the risk of developing breast cancer [77].

Glycylproline (Gly-Pro), a dipeptide that is typically an end-product of collagen metabolism, was significantly decreased in BCS vs HC. As noted, Gly and Pro have a high likelihood of being decreased in cancer patients. Alternatively, some neoplasia have reported increased proline content [78,79]. In the context of breast cancer, a study by Zareba et al. in MCF-7 breast cancer cells suggested that Gly-Pro and Gly-Pro-derived proline, along with the level of activity of the enzymes that cleave it (e.g., proline dehydrogenase/proline oxidase and prolidase) may play an intricate role in the balance between apoptosis, autophagy, and the growth of neoplastic cells [80]. Furthermore, in breast cancer, estrogen increases prolidase activity and collagen biosynthesis [81].

Variable importance plots (VIP) provide an estimation of the importance of each variable in the overall effect. The VIP plots for the untargeted plasma metabolomics showed that the Gly-Pro dipeptide was a highly important metabolite. The remaining metabolites found in the untargeted plasma metabolomics analysis, 8-hydroxyalanylclavam, and 12Z-Tetradecenyl acetate, have not previously been associated with cancer. The PCA results from both the untargeted urine and stool metabolomics showed a high overlap between the BCS and HC cohorts. No metabolites were significantly higher or lower in stool or urine samples when controlling for breast cancer type, age, and menopause status using multivariate modeling.

In the untargeted urinary metabolomics analysis, Model C showed that, in the subcohort of the BCS group who had received chemotherapy treatment, there was a set of 48 identifiable metabolites that were significantly lower (q < 0.1). Upon review of the available literature, we found only a small number of studies in breast cancer that have investigated urinary metabolomics and even fewer have included untargeted measures [82,83,84,85,86,87,88,89,90,91,92,93]. However, to our knowledge at the time of submission, this is the first of its kind study to investigate the untargeted urinary metabolome in a breast cancer survivor cohort that had received chemotherapy treatment. Given this fact, there are no datasets to compare the findings with and, thus, these findings warrant further investigation in a larger BCS cohort. This analysis was limited by the lack of power due to the small sample size (n: 10, 20% of BCS cohort).

### 4.6. Metagenomics

The microbiome sequencing put forth in this study provides valuable insight into the microbial composition of a BCS cohort. This gut microbial sequencing gives us the opportunity to assess the landscape of the gut metagenome of a relatively unassessed microbial population.

There was little association that could be drawn between our findings and those of other studies in the literature. This was mainly due to the depth of microbial analysis conducted in the present study and the scarcity of research in similar BCS cohorts. A study conducted by Smith et al. on the fecal microbial composition of BCS and their quality of life found that physical function, vitality, and mental health were negatively associated with *Ruminococcus* and *Dorea.* Additionally, they found that non-obese BCS had higher relative abundance of *Ruminococcus, Streptococcus, Roseburia,* and *Dorea* [12]. The present study found a slight increase in *Roseburia* and a decrease in *Streptococcus lutetiensis,* while the other results were not replicated.

BrCa patients who undergo chemotherapy treatment are at increased risk of developing metabolic disease and weight gain. This can potentially lead to increased morbidity and reduced quality of life in BCS. A study in BrCa patients with early-stage breast cancer compared the metagenome of patients who underwent adjuvant chemotherapy, adjuvant endocrine therapy, or both. They found that patients treated with chemotherapy only experienced clinically and statistically significant weight gain and fat distribution. Colonic inflammatory markers were also observed to have increased two-fold. Additionally, the researchers noted that these changes were accompanied by a reduction in the abundance and variety of microbial species in the gut microbiome [16]. The present study did not replicate this finding and, by most metrics, the cohorts were similar in terms of abundance and variety at a high level, with reported genus and species level differences.

In the study presented here, a simple analysis comparing BCS to HC showed multiple differences on each of the levels (species, genus, family, etc.) we investigated. This specific set of findings casts a wide net and can be leveraged in future research for translations into interventions that can be applied to breast cancer survivors. When controlling for age and menopause status as well as grouping by BrCa stage at diagnosis and BrCa subtype, a second, more resolved set of findings was illuminated, giving further resolution to these differential findings and their potential generalizability. Similarly, multiple correlations were found with other diseases known to affect the microbiome (such as irritable bowel syndrome, Parkinson’s disease, colorectal cancer, etc.). Utilizing these correlations could further open avenues to understanding significant changes in microbiome community structure and lead to novel interventions.

## 5. Conclusions, Future Directions, and Limitations

The present study was established as a hypothesis-seeking and hypothesis-generating investigation. When such multiomics studies are conducted, patterns of variance frequently emerge from the high dimensional data. These novel patterns of variance then form the basis of new hypotheses that can be followed up by future investigations. It is these novel hypotheses that frequently lead to treatment innovations.

It was acknowledged at the outset of the present study that treatment effects would be a confounding factor in the interpretation of how the biochemical characteristics being measured may be associated with disease development or treatment outcome. This is because treatment and time will naturally change the biochemical landscape. Nevertheless, this study has revealed several future research questions and has laid the groundwork for specific investigations. These can be considered for both newly diagnosed and breast cancer survivors.

First, this deep phenotyping study has led to a data set that can live as a molecular atlas of breast cancer survivors. This can serve as a frame of reference for future multiomics studies in a larger cohort of BCS, where the study will be more strongly powered by larger subject numbers. This may lead to the ability to detect signals that did not emerge from the current cohort size.

Second, the finding of altered omega-3 fatty acids warrants the exploration of fully quantitative omega-3 fatty acid status in the newly diagnosed and in BCS as a research effort. Since omega-3 status is clinically actionable, there is merit in examining omega-3 fatty acids status in the clinic. The provision of omega-3 dietary supplements could become a clinically justifiable intervention.

Third, examination of bile acids in the newly diagnosed and in a larger cohort of BCS is warranted. In particular, secondary bile acids are products of bioconversion by gut bacteria. When bile acids are released from the gut and distributed systemically, secondary bile acids may have clinical effects in tissues like the breast. Since secondary bile acids are modifiable by dietary inputs, such as prebiotics and probiotics, this path of investigation may one day lead to prophylactic or therapeutic food or dietary supplement interventions (primary or adjunctive). A future investigation of bile acids in the newly diagnosed would be justified.

Fourth, acylcarnitines were altered in this BCS cohort. In the BCS group, total carnitine was lower. Carnitine is a carrier molecule that transports fatty acids throughout the body. Variations in the types of fatty acids bound to carnitine can be highly informative as to how metabolism is changing. Moreover, carnitine also transports fatty acids across the mitochondrial membrane so they can be oxidized as an energy source via beta-oxidation. Thus, low total carnitine may be of clinical import in BCS, as it may adversely impact the ability to use fatty acids as an energy substrate necessary for the generation of ATP. Future work should investigate the acylcarnitine signature in the newly diagnosed to explore how this molecular class might influence disease progression and treatment outcomes. Additionally, carnitine status should be examined, as carnitine supplementation is widely used and can favorably impact energy metabolism. Further research should also explore whether this carnitine deficit is a result of declining endogenous carnitine synthesis, increased carnitine degradation, or reduced carnitine intake.

Fifth, the present study showed a strong genetic component of individual risk, evidenced by the divergence of polygenic risk scores between the HC and BCS cohorts. A future study in the newly diagnosed will have the ability to associate molecular characteristics with disease emergence and progression. Additionally, deep phenotyping study (using multiomics) in those with genetic risk (PRS), including asymptomatically, may reveal very early metabolic signatures (in those with genetic risk) that can lead to new treatment targets and preventive approaches. What this means is that molecular features like the transcriptome, proteome, metabolome, and microbiome (measured in this study) may reveal early metabolic signatures that influence the genetic risk to become manifest as a clinical disease.

Sixth, this study in BCS has allowed for the refinement of methods in multiomics sample collection, preanalytical processing, analysis, and interpretation. This refinement of multiomic methods in BCS is of notable value that can be deployed in larger studies of the newly diagnosed. A prospective multiomic study of the newly diagnosed can be a very important next step since this level of deep phenotyping has not been conducted in the newly diagnosed as of this writing. This would include assessment at the time of diagnosis and longitudinally during treatment, a study which has not to date been completed in a large cohort.

Seventh, it bears noting that a rudimentary analysis was performed on the time from treatment in this BCS cohort. In short, we looked for potential divergent signals between those who most recently completed treatment and those who were further out from completion of treatment. A limitation of this approach is that we did not have molecular data at the various time points in the time from treatment analysis. Future studies would seek to examine patients during the course of treatment in the newly diagnosed and follow them with multiomics analytics for a select number of years after treatment. This type of analysis would provide multiomic data at a time proximal to the time of diagnosis, during treatment, and post-treatment.

Eighth, the current study included a deep statistical analysis of the gut metagenome. However, due to the extensive amount of metagenomic data, future studies could expand the statistical analysis of the metagenome data from the existing study to examine additional signals. This may further clarify the biological meaning of the BCS cohort findings.

Finally, future investigations should pay careful attention to comorbidities, as the molecular and clinical phenotypes may stratify differently in accordance with these comorbidities.

## Figures and Tables

**Table 1:** Metadata: Description of the Cohort.

Metric ^1^		BCS	HC
Subjects (N)		50	50
Age, yrs (mean (SD))		62.8 (9.91)	63.2 (9.7)
BMI, kg/m^2^ (mean (SD))		28.8 (5.9)	27.5 (5)
Breast Cancer Type		Ductal: 35 (70%) Lobular: 12 (24%) Mixed: 3 (6%)	NA
Treatment Type (N (%))		Chemo: 10 (20%) Endo: 34 (68%) Radio: 32 (64%)	NA
Tamoxifen Use (N (%))		16 (32%)	NA
Breast Cancer Stage at Diagnosis (N (%))		Stage 0: 7 (14%) Stage I: 31 (62%) Stage II: 8 (16%) Stage III: 4 (8%)	NA
Type II Diabetes—Pre-Treatment		Yes: 5 (10%) No: 45 (90%)	NA
Type II Diabetes—Post-Treatment		Yes: 5 (10%) ^3^ No: 45 (90%)	NA
Type II Diabetes Health Cohort		NA	Yes: 6 (12%) No: 45 (88%)
HER2 BrCa Status		Negative: 40 (80%) Positive: 3 (6%) Unknown: 7 (14%)	NA
BRCA1 BrCa Status		Negative: 17 (34%) Pathogenic: 1 (2%) Not tested: 31 (62%) VUS: 1 (2%)	NA
BRCA2 BrCa Status		Negative: 17 (34%) Pathogenic: 1 (2%) Not tested: 31 (62%) VUS: 1 (2%)	NA
Menopause Status (N (%))		Pre: 8 (16%) Post: 42 (84%)	Pre: 6 (12%) Post: 44 (88%)
PHQ-8 Total Score (mean (SD))		1.58 (2.59)	1.06 (1.62)
GAD-7 Total Score (mean (SD))		1.16 (2.71)	1.30 (2.70)
Biotics (N (%))	Prebiotics	3 (6%)	1 (2%)
	Probiotics	1 (2%)	0 (0%)
	Antibiotics	2 (4%)	1 (2%)
History (N (%))	Diabetes	5 (10%)	6 (12%)
	Blood Pressure	14 (28%)	20 (40%)
	Depression	9 (18%)	5 (10%)
	Anxiety ^2^	12 (24%)	2 (4%)
	Pain	7 (14%)	3 (6%)
	Heart Problems	3 (6%)	3 (6%)

^1^ PHQ-8: Patient Health Questionnaire 8; GAD-7: Generalized Anxiety Disorder 7 questionnaire; Prebiotics: (“Are you currently taking prebiotic supplements?”); Probiotics: (“Are you currently taking probiotic supplements?); Antibiotics: (“Have you ever taken antibiotics for more than 3 months in the past year?); NA: not applicable; VUS: variant of unknown significance; BrCa: breast cancer. ^2^ Wilcoxon Rank-Sum Test, between-groups *p*-value < 0.05. ^3^ The participants with Type II diabetes (T2D) post-treatment were the same participants with T2D pre-treatment.

## Data Availability

The data presented in this study are available on request from the corresponding author. The data are not publicly available due to privacy or ethical restrictions.

## Appendix B

**Table A1:** Most Significant BrCa-specific PRS.

PGSa	Wilcox.p.Value	lm.Estimate	lm.p.Value	Wilcox.q.Value	lm.q.Value	Reported.Trait	Mapped.Trait.s...EFO.Label.
**PGS000511**	1.3 × 10^−7^	0.136170	3.4 × 10^−7^	4.7 × 10^−6^	4.9 × 10^−5^	Breast cancer (female)	breast carcinoma
**PGS000512**	2.4 × 10^−7^	0.503786	1.7 × 10^−7^	7.0 × 10^−8^	4.9 × 10^−5^	Breast cancer (female)	breast carcinoma
**PGS000510**	2.0 × 10^−7^	0.624025	6.3 × 10^−7^	5.8 × 10^−6^	6.0 × 10^−5^	Breast cancer (female)	breast carcinoma
**PGS000507**	4.7 × 10^−7^	0.144283	1.5 × 10^−6^	1.2 × 10^−5^	1.1 × 10^−4^	Breast cancer (female)	breast carcinoma
**PGS000008**	5.6 × 10^−7^	0.220445	8.7 × 10^−6^	1.3 × 10^−5^	5.0 × 10^−4^	ER-positive Breast Cancer	estrogen-receptor positive breast cancer
**PGS000504**	3.1 × 10^−8^	0.570125	1.2 × 10^−5^	1.5 × 10^−6^	5.9 × 10^−4^	Breast cancer (female)	breast carcinoma
**PGS000007**	3.6 × 10^−6^	0.199725	1.8 × 10^−5^	6.5 × 10^−5^	7.4 × 10^−4^	Breast Cancer	breast carcinoma
**PGS000536**	7.4 × 10^−9^	0.065889	2.4 × 10^−5^	5.3 × 10^−7^	8.6 × 10^−4^	Breast cancer (female)	breast carcinoma
**PGS003398**	1.2 × 10^−9^	0.197326	3.0 × 10^−5^	1.7 × 10^−7^	9.7 × 10^−4^	Breast Cancer	breast carcinoma
**PGS000531**	9.2 × 10^−8^	0.263083	3.7 × 10^−5^	3.7 × 10^−6^	1.0 × 10^−3^	Breast cancer (female)	breast carcinoma
**PGS000503**	6.9 × 10^−6^	0.157900	5.0 × 10^−5^	9.0 × 10^−5^	1.3 × 10^−3^	Breast cancer (female)	breast carcinoma
**PGS000539**	1.5 × 10^−7^	0.059395	5.4 × 10^−5^	4.7 × 10^−6^	1.3 × 10^−3^	Breast cancer (female)	breast carcinoma
**PGS000509**	5.9 × 10^−7^	0.624194	6.9 × 10^−5^	1.3 × 10^−5^	1.4 × 10^−3^	Breast cancer (female)	breast carcinoma
**PGS000528**	1.8 × 10^−6^	0.081497	6.8 × 10^−5^	3.8 × 10^−5^	1.4 × 10^−3^	Breast cancer (female)	breast carcinoma
**PGS000508**	6.0 × 10^−6^	0.149773	7.4 × 10^−5^	9.0 × 10^−5^	1.4 × 10^−3^	Breast cancer (female)	breast carcinoma
**PGS000873**	1.8 × 10^−9^	0.193709	1.0 × 10^−4^	1.7 × 10^−7^	1.8 × 10^−3^	Breast cancer	breast carcinoma
**PGS000052**	1.6 × 10^−8^	0.201100	2.4 × 10^−4^	9.3 × 10^−7^	4.2 × 10^−3^	Breast cancer	breast carcinoma
**PGS000535**	9.8 × 10^−5^	0.061701	3.5 × 10^−4^	7.6 × 10^−4^	5.4 × 10^−3^	Breast cancer (female)	breast carcinoma
**PGS003380**	1.7 × 10^−5^	0.389549	3.4 × 10^−4^	2.0 × 10^−4^	5.4 × 10^−3^	Breast cancer	breast carcinoma
**PGS000527**	3.1 × 10^−5^	0.078368	3.8 × 10^−4^	3.5 × 10^−4^	5.5 × 10^−3^	Breast cancer (female)	breast carcinoma
**PGS000214**	3.0 × 10^−6^	0.269912	5.9 × 10^−4^	5.9 × 10^−5^	7.8 × 10^−3^	Breast cancer intrinsic-like subtype (luminal B-like)	luminal B breast carcinoma
**PGS000497**	6.2 × 10^−5^	0.321878	7.0 × 10^−4^	5.5 × 10^−4^	7.8 × 10^−3^	Breast cancer (female)	breast carcinoma
**PGS000498**	6.2 × 10^−5^	0.321878	7.0 × 10^−4^	5.5 × 10^−4^	7.8 × 10^−3^	Breast cancer (female)	breast carcinoma
**PGS000502**	8.0 × 10^−5^	0.489076	6.6 × 10^−4^	6.6 × 10^−4^	7.8 × 10^−3^	Breast cancer (female)	breast carcinoma
**PGS000505**	4.1 × 10^−6^	0.383257	7.0 × 10^−4^	6.6 × 10^−5^	7.8 × 10^−3^	Breast cancer (female)	breast carcinoma
**PGS000506**	4.1 × 10^−6^	0.383257	7.0 × 10^−4^	6.6 × 10^−5^	7.8 × 10^−3^	Breast cancer (female)	breast carcinoma
**PGS000472**	3.7 × 10^−4^	0.007524	7.5 × 10^−4^	2.3 × 10^−3^	8.0 × 10^−3^	Breast cancer (female)	breast carcinoma
**PGS000335**	1.0 × 10^−5^	0.148391	8.3 × 10^−4^	1.2 × 10^−4^	8.5 × 10^−3^	Breast cancer	breast carcinoma
**PGS000499**	6.9 × 10^−5^	0.163507	1.2 × 10^−3^	6.0 × 10^−4^	0.011942	Breast cancer (female)	breast carcinoma
**PGS000501**	5.7 × 10^−5^	0.491264	1.2 × 10^−3^	5.5 × 10^−4^	0.011942	Breast cancer (female)	breast carcinoma
**PGS000002**	6.8 × 10^−6^	0.141149	1.3 × 10^−3^	9.0 × 10^−5^	0.012108	ER-positive Breast Cancer	estrogen-receptor positive breast cancer
**PGS000540**	1.2 × 10^−5^	0.030463	1.3 × 10^−3^	1.4 × 10^−4^	0.012108	Breast cancer (female)	breast carcinoma
**PGS000212**	7.8 × 10^−5^	0.267451	1.3 × 10^−3^	6.6 × 10^−4^	0.012123	Breast cancer intrinsic-like subtype (luminal A-like)	luminal A breast carcinoma
**PGS000213**	6.3 × 10^−6^	0.217897	2.0 × 10^−3^	9.0 × 10^−5^	0.017031	Breast cancer intrinsic-like subtype (luminal B/HER2-negative-like)	HER2-negative breast carcinoma
**PGS000001**	4.2 × 10^−4^	0.128408	2.3 × 10^−3^	2.5 × 10^−3^	0.017187	Breast Cancer	breast carcinoma
**PGS000004**	2.9 × 10^−3^	0.192750	2.5 × 10^−3^	1.4 × 10^−2^	0.017187	Breast Cancer	breast carcinoma
**PGS000005**	2.0 × 10^−3^	0.204576	2.2 × 10^−3^	1.0 × 10^−2^	0.017187	ER-positive Breast Cancer	estrogen-receptor positive breast cancer
**PGS000332**	4.3 × 10^−4^	0.136928	2.1 × 10^−3^	2.5 × 10^−3^	0.017187	Breast cancer	breast carcinoma
**PGS000344**	5.8 × 10^−5^	0.190588	2.5 × 10^−3^	5.5 × 10^−4^	0.017187	Breast cancer	breast carcinoma
**PGS000473**	1.2 × 10^−4^	0.038474	2.5 × 10^−3^	9.3 × 10^−4^	0.017187	Breast cancer (female)	breast carcinoma
**PGS000480**	1.0 × 10^−3^	0.007254	2.5 × 10^−3^	5.5 × 10^−3^	0.017187	Breast cancer (female)	breast carcinoma
**PGS000533**	5.1 × 10^−5^	0.200274	2.3 × 10^−3^	5.3 × 10^−4^	0.017187	Breast cancer (female)	breast carcinoma
**PGS000534**	5.1 × 10^−5^	0.200274	2.3 × 10^−3^	5.3 × 10^−4^	0.017187	Breast cancer (female)	breast carcinoma
**PGS000347**	1.5 × 10^−4^	0.197233	2.6 × 10^−3^	1.1 × 10^−3^	0.017434	Estrogen receptor-positive breast cancer	estrogen-receptor positive breast cancer
**PGS000009**	4.4 × 10^−4^	0.143606	2.8 × 10^−3^	2.5 × 10^−3^	0.018270	ER-negative Breast Cancer	estrogen-receptor-negative breast cancer
**PGS000538**	1.3 × 10^−4^	0.182262	2.9 × 10^−3^	1.0 × 10^−3^	0.018555	Breast cancer (female)	breast carcinoma
**PGS000500**	2.4 × 10^−4^	0.172722	3.8 × 10^−3^	1.6 × 10^−3^	0.023371	Breast cancer (female)	breast carcinoma
**PGS000046**	8.0 × 10^−4^	0.132093	5.6 × 10^−3^	4.5 × 10^−3^	0.033573	Estrogen receptor [ER]-positive breast cancer	estrogen-receptor positive breast cancer
**PGS000045**	1.8 × 10^−4^	0.117902	6.0 × 10^−3^	1.2 × 10^−3^	0.034689	Breast cancer	breast carcinoma
**PGS003399**	2.1 × 10^−4^	0.192520	6.0 × 10^−3^	1.4 × 10^−3^	0.034689	Breast Cancer	breast carcinoma

## Appendix F

**Table A2:** Metabolite Findings in Model C, variable TxChemo. Controlled for ~BrCa + Age + Menopause + TxTamoxifen + TxChemo + TxEndo + TxRadio.

Metabolite (MS Mode)	Variable	Estimate	p.Value	Qval
Lys Pro (C18+)	TxChemoY	−1.466	2.11 × 10^−06^	0.0189
L-Methionine S-oxide (hilic+)	TxChemoY	−1.145	4.43 × 10^−06^	0.0199
Chlorfenprop-methyl (hilic−)	TxChemoY	−1.028	1.06 × 10^−05^	0.0237
Maleamic acid (C18−)	TxChemoY	−1.123	1.67 × 10^−05^	0.0299
Homocysteinesulfinic acid (C18+)	TxChemoY	−0.739	2.72 × 10^−05^	0.0349
N-Feruloylglycine (hilic−)	TxChemoY	−3.114	2.75 × 10^−05^	0.0349
AG-041R (hilic−)	TxChemoY	−2.777	5.92 × 10^−05^	0.0497
4-methylthiazole-5-acetic-acid (hilic+)	TxChemoY	−2.145	6.09 × 10^−05^	0.0497
4-Sulfobenzoate (C18−)	TxChemoY	−1.236	8.45 × 10^−05^	0.0566
Caprylic acid (C18−)	TxChemoY	−1.347	9.57 × 10^−05^	0.0566
SC-58125 (C18−)	TxChemoY	−2.573	1.02 × 10^−04^	0.0566
N-Methyl-2-oxoglutaramate (C18−)	TxChemoY	−0.363	1.17 × 10^−04^	0.0566
Lewis a trisaccharide (C18−)	TxChemoY	−0.367	1.18 × 10^−04^	0.0566
Lovastatin (hilic+)	TxChemoY	−2.541	1.24 × 10^−04^	0.0566
N-Acetylneuraminic Acid (hilic−)	TxChemoY	−2.248	1.28 × 10^−04^	0.0566
3-Keto-scyllo-inosamine (C18+)	TxChemoY	−0.676	1.31 × 10^−04^	0.0566
Carboxymethyloxysuccinate (C18+)	TxChemoY	−0.831	1.36 × 10^−04^	0.0566
4-(Trimethylammonio)but-2-enoate (hilic+)	TxChemoY	−0.500	1.39 × 10^−04^	0.0566
9alpha-Fluoro-6alpha-methylprednisolone 21-acetate (C18+)	TxChemoY	−2.617	1.65 × 10^−04^	0.0599
5-Ethyl-5-(1-methyl-3-carboxypropyl)barbituric acid (hilic+)	TxChemoY	−0.283	1.68 × 10^−04^	0.0599
Pivalic acid (C18−)	TxChemoY	−0.758	1.91 × 10^−04^	0.0604
Lys-Trp-OH (C18−)	TxChemoY	−0.758	1.99 × 10^−04^	0.0604
4-Sulfobenzoate (hilic−)	TxChemoY	−0.263	2.02 × 10^−04^	0.0604
Aldosterone 18-glucuronide (C18+)	TxChemoY	−2.057	2.20 × 10^−04^	0.0636
L-Glutamic acid n-butyl ester (C18−)	TxChemoY	−1.897	2.63 × 10^−04^	0.0700
Dopamine 3-O-sulfate (C18−)	TxChemoY	−0.790	2.65 × 10^−04^	0.0700
Homolanthionine (hilic−)	TxChemoY	−1.997	3.03 × 10^−04^	0.0777
N-Acetylvanilalanine (C18−)	TxChemoY	−1.691	3.32 × 10^−04^	0.0782
5-aminosalicyluric acid (hilic−)	TxChemoY	−1.017	3.37 × 10^−04^	0.0782
a-hydroxyisovalerate (C18−)	TxChemoY	−1.734	3.51 × 10^−04^	0.0782
Propanoic acid, 2-hydroxy-3-[(4-hydroxy-1-naphthalenyl)oxy]- (C18−)	TxChemoY	−0.763	3.62 × 10^−04^	0.0782
8-Hydroxyondansetron glucuronide (hilic+)	TxChemoY	−0.724	3.64 × 10^−04^	0.0782
2-Amino-5-oxohexanoate (C18+)	TxChemoY	−1.639	4.07 × 10^−04^	0.0837
Octanoic acid, 3-amino-, (1)- (C18+)	TxChemoY	−1.664	4.30 × 10^−04^	0.0837
Cardiogenol C (hilic+)	TxChemoY	−0.238	4.36 × 10^−04^	0.0837
Betaine (hilic+)	TxChemoY	−0.234	4.37 × 10^−04^	0.0837
2,4-Dichlorophenoxybutyric Acid (C18−)	TxChemoY	−0.353	4.39 × 10^−04^	0.0837
2-Napthyloxyacetic acid (hilic−)	TxChemoY	−0.687	4.51 × 10^−04^	0.0843
Ripazepam (C18+)	TxChemoY	−0.809	4.64 × 10^−04^	0.0850
L-glycyl-L-hydroxyproline (hilic+)	TxChemoY	−0.641	5.25 × 10^−04^	0.0942
2,3-Dihydroxynaphthalene (C18+)	TxChemoY	−0.809	5.52 × 10^−04^	0.0968
Clitidine (hilic+)	TxChemoY	−0.221	5.62 × 10^−04^	0.0968
N2-Acetyl-L-aminoadipate (hilic+)	TxChemoY	−0.738	5.90 × 10^−04^	0.0968
(2S,3S)-2-hydroxytridecane-1,2,3-tricarboxylic acid (hilic+)	TxChemoY	−2.588	5.93 × 10^−04^	0.0968
Ursodeoxycholic acid 3-sulfate (C18−)	TxChemoY	−1.143	6.04 × 10^−04^	0.0968
beta-Hydroxyacteoside (C18−)	TxChemoY	−1.497	6.13 × 10^−04^	0.0968
L-prolyl-L-proline (C18+)	TxChemoY	−0.471	6.21 × 10^−04^	0.0968
Tyr Val Trp (hilic−)	TxChemoY	−2.168	6.36 × 10^−04^	0.0968
4-Sulfobenzoate (C18−)	TxChemoY	−1.522	6.37 × 10^−04^	0.0968
DL-Cycloserine (hilic+)	TxChemoY	−0.248	6.50 × 10^−04^	0.0971

## References

[1] Sung H., Ferlay J., Siegel R.L., Laversanne M., Soerjomataram I., Jemal A., Bray F. (2021). Global Cancer Statistics 2020: GLOBOCAN Estimates of Incidence and Mortality Worldwide for 36 Cancers in 185 Countries. CA A Cancer J. Clin..

[2] Siegel R.L., Miller K.D., Wagle N.S., Jemal A. (2023). Cancer Statistics, 2023. CA A Cancer J. Clin..

[3] An R., Yu H., Wang Y., Lu J., Gao Y., Xie X., Zhang J. (2022). Integrative Analysis of Plasma Metabolomics and Proteomics Reveals the Metabolic Landscape of Breast Cancer. Cancer Metab..

[4] Barupal D.K., Gao B., Budczies J., Phinney B.S., Perroud B., Denkert C., Fiehn O. (2019). Prioritization of Metabolic Genes as Novel Therapeutic Targets in Estrogen-Receptor Negative Breast Tumors Using Multi-Omics Data and Text Mining. Oncotarget.

[5] Bellerba F., Chatziioannou A.C., Jasbi P., Robinot N., Keski-Rahkonen P., Trolat A., Vozar B., Hartman S.J., Scalbert A., Bonanni B. (2022). Metabolomic Profiles of Metformin in Breast Cancer Survivors: A Pooled Analysis of Plasmas from Two Randomized Placebo-Controlled Trials. J. Transl. Med..

[6] Debik J., Euceda L.R., Lundgren S., von der Lippe Gythfeldt H., Garred Ø., Borgen E., Engebraaten O., Bathen T.F., Giskeødegård G.F. (2019). Assessing Treatment Response and Prognosis by Serum and Tissue Metabolomics in Breast Cancer Patients. J. Proteome Res..

[7] Dowling P., Henry M., Meleady P., Clarke C., Gately K., O’Byrne K., Connolly E., Lynch V., Ballot J., Gullo G. (2015). Metabolomic and Proteomic Analysis of Breast Cancer Patient Samples Suggests That Glutamate and 12-HETE in Combination with CA15-3 May Be Useful Biomarkers Reflecting Tumour Burden. Metabolomics.

[8] Hassan M.A., Al-Sakkaf K., Shait Mohammed M.R., Dallol A., Al-Maghrabi J., Aldahlawi A., Ashoor S., Maamra M., Ragoussis J., Wu W. (2020). Integration of Transcriptome and Metabolome Provides Unique Insights to Pathways Associated With Obese Breast Cancer Patients. Front. Oncol..

[9] Haukaas T.H., Euceda L.R., Giskeødegård G.F., Lamichhane S., Krohn M., Jernström S., Aure M.R., Lingjærde O.C., Schlichting E., Garred Ø. (2016). Metabolic Clusters of Breast Cancer in Relation to Gene- and Protein Expression Subtypes. Cancer Metab..

[10] Huang S., Chong N., Lewis N.E., Jia W., Xie G., Garmire L.X. (2016). Novel Personalized Pathway-Based Metabolomics Models Reveal Key Metabolic Pathways for Breast Cancer Diagnosis. Genome Med..

[11] Luo X., Yu H., Song Y., Sun T. (2019). Integration of Metabolomic and Transcriptomic Data Reveals Metabolic Pathway Alteration in Breast Cancer and Impact of Related Signature on Survival. J. Cell. Physiol..

[12] Smith K.S., Tissier A., Bail J.R., Novak J.R., Morrow C.D., Demark-Wahnefried W., Frugé A.D. (2022). Health-Related Quality of Life Is Associated with Fecal Microbial Composition in Breast Cancer Survivors. Support. Care Cancer.

[13] Starodubtseva N.L., Tokareva A.O., Rodionov V.V., Brzhozovskiy A.G., Bugrova A.E., Chagovets V.V., Kometova V.V., Kukaev E.N., Soares N.C., Kovalev G.I. (2023). Integrating Proteomics and Lipidomics for Evaluating the Risk of Breast Cancer Progression: A Pilot Study. Biomedicines.

[14] Tang X., Lin C.-C., Spasojevic I., Iversen E.S., Chi J.-T., Marks J.R. (2014). A Joint Analysis of Metabolomics and Genetics of Breast Cancer. Breast Cancer Res..

[15] Terunuma A., Putluri N., Mishra P., Mathé E.A., Dorsey T.H., Yi M., Wallace T.A., Issaq H.J., Zhou M., Killian J.K. (2014). MYC-Driven Accumulation of 2-Hydroxyglutarate Is Associated with Breast Cancer Prognosis. J. Clin. Investig..

[16] Walker J., Joy A.A., Vos L.J., Stenson T.H., Mackey J.R., Jovel J., Kao D., Madsen K.L., Wong G.K.-S. (2023). Chemotherapy-Induced Weight Gain in Early-Stage Breast Cancer: A Prospective Matched Cohort Study Reveals Associations with Inflammation and Gut Dysbiosis. BMC Med..

[17] Xiao Y., Ma D., Yang Y.-S., Yang F., Ding J.-H., Gong Y., Jiang L., Ge L.-P., Wu S.-Y., Yu Q. (2022). Comprehensive Metabolomics Expands Precision Medicine for Triple-Negative Breast Cancer. Cell Res..

[18] Meydan C., Afshinnekoo E., Rickard N., Daniels G., Kunces L., Hardy T., Lili L., Pesce S., Jacobson P., Mason C.E. (2020). Improved Gastrointestinal Health for Irritable Bowel Syndrome with Metagenome-Guided Interventions. Precis. Clin. Med..

[19] Li H., Durbin R. (2009). Fast and Accurate Short Read Alignment with Burrows-Wheeler Transform. Bioinformatics.

[20] Kendig K.I., Baheti S., Bockol M.A., Drucker T.M., Hart S.N., Heldenbrand J.R., Hernaez M., Hudson M.E., Kalmbach M.T., Klee E.W. (2019). Sentieon DNASeq Variant Calling Workflow Demonstrates Strong Computational Performance and Accuracy. Front. Genet..

[21] Freed D., Pan R., Chen H., Li Z., Hu J., Aldana R. (2022). DNAscope: High Accuracy Small Variant Calling Using Machine Learning. bioRxiv.

[22] Mills R.E., Luttig C.T., Larkins C.E., Beauchamp A., Tsui C., Pittard W.S., Devine S.E. (2006). An Initial Map of Insertion and Deletion (INDEL) Variation in the Human Genome. Genome Res..

[23] Sherry S.T., Ward M., Sirotkin K. (1999). dbSNP-Database for Single Nucleotide Polymorphisms and Other Classes of Minor Genetic Variation. Genome Res..

[24] Lambert S.A., Gil L., Jupp S., Ritchie S.C., Xu Y., Buniello A., McMahon A., Abraham G., Chapman M., Parkinson H. (2021). The Polygenic Score Catalog as an Open Database for Reproducibility and Systematic Evaluation. Nat. Genet..

[25] Wainberg M., Magis A.T., Earls J.C., Lovejoy J.C., Sinnott-Armstrong N., Omenn G.S., Hood L., Price N.D. (2020). Multiomic Blood Correlates of Genetic Risk Identify Presymptomatic Disease Alterations. Proc. Natl. Acad. Sci. USA.

[26] Gold L., Ayers D., Bertino J., Bock C., Bock A., Brody E.N., Carter J., Dalby A.B., Eaton B.E., Fitzwater T. (2010). Aptamer-Based Multiplexed Proteomic Technology for Biomarker Discovery. PLoS ONE.

[27] Kim C.H., Tworoger S.S., Stampfer M.J., Dillon S.T., Gu X., Sawyer S.J., Chan A.T., Libermann T.A., Eliassen A.H. (2018). Stability and Reproducibility of Proteomic Profiles Measured with an Aptamer-Based Platform. Sci. Rep..

[28] Van der Maaten L., Hinton G. (2008). Visualizing Data Using T-SNE. J. Mach. Learn. Res..

[29] Bolger A.M., Lohse M., Usadel B. (2014). Trimmomatic: A Flexible Trimmer for Illumina Sequence Data. Bioinformatics.

[30] Breitwieser F.P., Baker D.N., Salzberg S.L. (2018). KrakenUniq: Confident and Fast Metagenomics Classification Using Unique k-Mer Counts. Genome Biol..

[31] Lu J., Breitwieser F.P., Thielen P., Salzberg S.L. (2016). Bracken: Estimating Species Abundance in Metagenomics Data. PeerJ Comput. Sci..

[32] Beghini F., McIver L.J., Blanco-Míguez A., Dubois L., Asnicar F., Maharjan S., Mailyan A., Manghi P., Scholz M., Thomas A.M. (2021). Integrating Taxonomic, Functional, and Strain-Level Profiling of Diverse Microbial Communities with bioBakery 3. eLife.

[33] Chen J., Bittinger K., Charlson E.S., Hoffmann C., Lewis J., Wu G.D., Collman R.G., Bushman F.D., Li H. (2012). Associating Microbiome Composition with Environmental Covariates Using Generalized UniFrac Distances. Bioinformatics.

[34] Bray J.R., Curtis J.T. (1957). An Ordination of the Upland Forest Communities of Southern Wisconsin. Ecol. Monogr..

[35] Mallick H., Rahnavard A., McIver L.J., Ma S., Zhang Y., Nguyen L.H., Tickle T.L., Weingart G., Ren B., Schwager E.H. (2021). Multivariable Association Discovery in Population-Scale Meta-Omics Studies. PLoS Comput. Biol..

[36] Robinson M.D., Oshlack A. (2010). A Scaling Normalization Method for Differential Expression Analysis of RNA-Seq Data. Genome Biol..

[37] Janssens Y., Nielandt J., Bronselaer A., Debunne N., Verbeke F., Wynendaele E., Van Immerseel F., Vandewynckel Y.-P., De Tré G., De Spiegeleer B. (2018). Disbiome Database: Linking the Microbiome to Disease. BMC Microbiol..

[38] Gunn S., Wainberg M., Song Z., Andersen S., Boudreau R., Feitosa M.F., Tan Q., Montasser M.E., O’Connell J.R., Stitziel N. (2022). Distribution of 54 Polygenic Risk Scores for Common Diseases in Long Lived Individuals and Their Offspring. Geroscience.

[39] Roberts E., Howell S., Evans D.G. (2023). Polygenic Risk Scores and Breast Cancer Risk Prediction. Breast.

[40] Gjerde J., Geisler J., Lundgren S., Ekse D., Varhaug J.E., Mellgren G., Steen V.M., Lien E.A. (2010). Associations between Tamoxifen, Estrogens, and FSH Serum Levels during Steady State Tamoxifen Treatment of Postmenopausal Women with Breast Cancer. BMC Cancer.

[41] Zhou J., Chen Y., Huang Y., Long J., Wan F., Zhang S. (2013). Serum Follicle-Stimulating Hormone Level Is Associated with Human Epidermal Growth Factor Receptor Type 2 and Ki67 Expression in Post-Menopausal Females with Breast Cancer. Oncol. Lett..

[42] Sherbet G.V., Cajone F. (2005). Stathmin in Cell Proliferation and Cancer Progression. Cancer Genom. Proteom..

[43] Kuang X.-Y., Jiang H.-S., Li K., Zheng Y.-Z., Liu Y.-R., Qiao F., Li S., Hu X., Shao Z.-M. (2016). The Phosphorylation-Specific Association of STMN1 with GRP78 Promotes Breast Cancer Metastasis. Cancer Lett..

[44] Askeland C., Wik E., Finne K., Birkeland E., Arnes J.B., Collett K., Knutsvik G., Krüger K., Davidsen B., Aas T. (2020). Stathmin Expression Associates with Vascular and Immune Responses in Aggressive Breast Cancer Subgroups. Sci. Rep..

[45] Xie Z., Zhen T., Lin Y., Shao N., Kuang X. (2022). The Prognostic Role of a Phospho-Stathmin 1 Signature in Breast Cancer Treated with Neoadjuvant Chemotherapy. Gland. Surg..

[46] Dingjan I., Linders P.T.A., Verboogen D.R.J., Revelo N.H., ter Beest M., van den Bogaart G. (2018). Endosomal and Phagosomal SNAREs. Physiol. Rev..

[47] Parveen S., Khamari A., Raju J., Coppolino M.G., Datta S. (2022). Syntaxin 7 Contributes to Breast Cancer Cell Invasion by Promoting Invadopodia Formation. J. Cell Sci..

[48] Maniam S., Maniam S. (2021). Small Molecules Targeting Programmed Cell Death in Breast Cancer Cells. Int. J. Mol. Sci..

[49] Luo H., Qin Y., Reu F., Ye S., Dai Y., Huang J., Wang F., Zhang D., Pan L., Zhu H. (2016). Microarray-Based Analysis and Clinical Validation Identify Ubiquitin-Conjugating Enzyme E2E1 (UBE2E1) as a Prognostic Factor in Acute Myeloid Leukemia. J. Hematol. Oncol..

[50] Desai S.D., Reed R.E., Burks J., Wood L.M., Pullikuth A.K., Haas A.L., Liu L.F., Breslin J.W., Meiners S., Sankar S. (2012). ISG15 Disrupts Cytoskeletal Architecture and Promotes Motility in Human Breast Cancer Cells. Exp. Biol. Med..

[51] Du X., Song H., Shen N., Hua R., Yang G. (2021). The Molecular Basis of Ubiquitin-Conjugating Enzymes (E2s) as a Potential Target for Cancer Therapy. Int. J. Mol. Sci..

[52] Voutsadakis I.A. (2013). Ubiquitin- and Ubiquitin-like Proteins-Conjugating Enzymes (E2s) in Breast Cancer. Mol. Biol. Rep..

[53] Picon-Ruiz M., Morata-Tarifa C., Valle-Goffin J.J., Friedman E.R., Slingerland J.M. (2017). Obesity and Adverse Breast Cancer Risk and Outcome: Mechanistic Insights and Strategies for Intervention. CA Cancer J. Clin..

[54] Mihalik S.J., Goodpaster B.H., Kelley D.E., Chace D.H., Vockley J., Toledo F.G.S., DeLany J.P. (2010). Increased Levels of Plasma Acylcarnitines in Obesity and Type 2 Diabetes and Identification of a Marker of Glucolipotoxicity. Obesity.

[55] Shen J., Yan L., Liu S., Ambrosone C.B., Zhao H. (2013). Plasma Metabolomic Profiles in Breast Cancer Patients and Healthy Controls: By Race and Tumor Receptor Subtypes. Transl. Oncol..

[56] Ozmen H.K., Erdemci B., Askin S., Sezen O. (2017). Carnitine and Adiponectin Levels in Breast Cancer after Radiotherapy. Open Med..

[57] Režen T., Rozman D., Kovács T., Kovács P., Sipos A., Bai P., Mikó E. (2022). The Role of Bile Acids in Carcinogenesis. Cell. Mol. Life Sci..

[58] Giordano C., Catalano S., Panza S., Vizza D., Barone I., Bonofiglio D., Gelsomino L., Rizza P., Fuqua S.A.W., Andò S. (2011). Farnesoid X Receptor Inhibits Tamoxifen-Resistant MCF-7 Breast Cancer Cell Growth through Downregulation of HER2 Expression. Oncogene.

[59] Journe F., Durbecq V., Chaboteaux C., Rouas G., Laurent G., Nonclercq D., Sotiriou C., Body J.-J., Larsimont D. (2009). Association between Farnesoid X Receptor Expression and Cell Proliferation in Estrogen Receptor-Positive Luminal-like Breast Cancer from Postmenopausal Patients. Breast Cancer Res. Treat..

[60] Luo C., Zhang X., He Y., Chen H., Liu M., Wang H., Tang L., Tu G., Ding M. (2021). A Pseudo-Targeted Metabolomics Study Based on Serum Bile Acids Profiling for the Differential Diagnosis of Benign and Malignant Breast Lesions. Steroids.

[61] Costarelli V., Sanders T.A.B. (2002). Plasma Deoxycholic Acid Concentration Is Elevated in Postmenopausal Women with Newly Diagnosed Breast Cancer. Eur. J. Clin. Nutr..

[62] Wang N., Yang J., Han W., Han M., Liu X., Jiang L., Cao H., Jing M., Sun T., Xu J. (2022). Identifying Distinctive Tissue and Fecal Microbial Signatures and the Tumor-Promoting Effects of Deoxycholic Acid on Breast Cancer. Front. Cell. Infect. Microbiol..

[63] Tang W., Putluri V., Ambati C.R., Dorsey T.H., Putluri N., Ambs S. (2019). Liver- and Microbiome-Derived Bile Acids Accumulate in Human Breast Tumors and Inhibit Growth and Improve Patient Survival. Clin. Cancer Res..

[64] Cala M.P., Aldana J., Medina J., Sánchez J., Guio J., Wist J., Meesters R.J.W. (2018). Multiplatform Plasma Metabolic and Lipid Fingerprinting of Breast Cancer: A Pilot Control-Case Study in Colombian Hispanic Women. PLoS ONE.

[65] Pakiet A., Jędrzejewska A., Duzowska K., Wacławska A., Jabłońska P., Zieliński J., Mika A., Śledziński T., Słomińska E. (2023). Serum Fatty Acid Profiles in Breast Cancer Patients Following Treatment. BMC Cancer.

[66] Xu S., Chen T., Dong L., Li T., Xue H., Gao B., Ding X., Wang H., Li H. (2020). Fatty Acid Synthase Promotes Breast Cancer Metastasis by Mediating Changes in Fatty Acid Metabolism. Oncol. Lett..

[67] Hidaka B.H., Li S., Harvey K.E., Carlson S.E., Sullivan D.K., Kimler B.F., Zalles C.M., Fabian C.J. (2015). Omega-3 and Omega-6 Fatty Acids in Blood and Breast Tissue of High-Risk Women and Association with Atypical Cytomorphology. Cancer Prev. Res..

[68] Fabian C.J., Kimler B.F., Hursting S.D. (2015). Omega-3 Fatty Acids for Breast Cancer Prevention and Survivorship. Breast Cancer Res..

[69] Nindrea R.D., Aryandono T., Lazuardi L., Dwiprahasto I. (2019). Association of Dietary Intake Ratio of N-3/n-6 Polyunsaturated Fatty Acids with Breast Cancer Risk in Western and Asian Countries: A Meta-Analysis. Asian Pac. J. Cancer Prev..

[70] Yang B., Ren X.-L., Fu Y.-Q., Gao J.-L., Li D. (2014). Ratio of N-3/n-6 PUFAs and Risk of Breast Cancer: A Meta-Analysis of 274135 Adult Females from 11 Independent Prospective Studies. BMC Cancer.

[71] Bel’skaya L.V., Gundyrev I.A., Solomatin D.V. (2023). The Role of Amino Acids in the Diagnosis, Risk Assessment, and Treatment of Breast Cancer: A Review. Curr. Issues Mol. Biol..

[72] Lai H.-S., Lee J.-C., Lee P.-H., Wang S.-T., Chen W.-J. (2005). Plasma Free Amino Acid Profile in Cancer Patients. Semin. Cancer Biol..

[73] Yang L., Wang Y., Cai H., Wang S., Shen Y., Ke C. (2020). Application of Metabolomics in the Diagnosis of Breast Cancer: A Systematic Review. J. Cancer.

[74] Jobard E., Dossus L., Baglietto L., Fornili M., Lécuyer L., Mancini F.R., Gunter M.J., Trédan O., Boutron-Ruault M.-C., Elena-Herrmann B. (2021). Investigation of Circulating Metabolites Associated with Breast Cancer Risk by Untargeted Metabolomics: A Case–Control Study Nested within the French E3N Cohort. Br. J. Cancer.

[75] Lécuyer L., Victor Bala A., Deschasaux M., Bouchemal N., Nawfal Triba M., Vasson M.-P., Rossary A., Demidem A., Galan P., Hercberg S. (2018). NMR Metabolomic Signatures Reveal Predictive Plasma Metabolites Associated with Long-Term Risk of Developing Breast Cancer. Int. J. Epidemiol..

[76] Stevens V.L., Carter B.D., Jacobs E.J., McCullough M.L., Teras L.R., Wang Y. (2023). A Prospective Case–Cohort Analysis of Plasma Metabolites and Breast Cancer Risk. Breast Cancer Res..

[77] His M., Viallon V., Dossus L., Gicquiau A., Achaintre D., Scalbert A., Ferrari P., Romieu I., Onland-Moret N.C., Weiderpass E. (2019). Prospective Analysis of Circulating Metabolites and Breast Cancer in EPIC. BMC Med..

[78] Catchpole G., Platzer A., Weikert C., Kempkensteffen C., Johannsen M., Krause H., Jung K., Miller K., Willmitzer L., Selbig J. (2011). Metabolic Profiling Reveals Key Metabolic Features of Renal Cell Carcinoma. J. Cell. Mol. Med..

[79] Hirayama A., Kami K., Sugimoto M., Sugawara M., Toki N., Onozuka H., Kinoshita T., Saito N., Ochiai A., Tomita M. (2009). Quantitative Metabolome Profiling of Colon and Stomach Cancer Microenvironment by Capillary Electrophoresis Time-of-Flight Mass Spectrometry. Cancer Res..

[80] Zareba I., Celinska-Janowicz K., Surazynski A., Miltyk W., Palka J. (2018). Proline Oxidase Silencing Induces Proline-Dependent pro-Survival Pathways in MCF-7 Cells. Oncotarget.

[81] Lewoniewska S., Oscilowska I., Forlino A., Palka J. (2021). Understanding the Role of Estrogen Receptor Status in PRODH/POX-Dependent Apoptosis/Survival in Breast Cancer Cells. Biology.

[82] Subramani R., Poudel S., Smith K.D., Estrada A., Lakshmanaswamy R. (2022). Metabolomics of Breast Cancer: A Review. Metabolites.

[83] Nam H., Chung B.C., Kim Y., Lee K., Lee D. (2009). Combining Tissue Transcriptomics and Urine Metabolomics for Breast Cancer Biomarker Identification. Bioinformatics.

[84] Yang M., Jiang J., Hua L., Jiang D., Wang Y., Li D., Wang R., Zhang X., Li H. (2023). Rapid Detection of Volatile Organic Metabolites in Urine by High-Pressure Photoionization Mass Spectrometry for Breast Cancer Screening: A Pilot Study. Metabolites.

[85] Slupsky C.M., Steed H., Wells T.H., Dabbs K., Schepansky A., Capstick V., Faught W., Sawyer M.B. (2010). Urine Metabolite Analysis Offers Potential Early Diagnosis of Ovarian and Breast Cancers. Clin. Cancer Res..

[86] Cala M., Aldana J., Sánchez J., Guio J., Meesters R.J.W. (2018). Urinary Metabolite and Lipid Alterations in Colombian Hispanic Women with Breast Cancer: A Pilot Study. J. Pharm. Biomed. Anal..

[87] Woo H.M., Kim K.M., Choi M.H., Jung B.H., Lee J., Kong G., Nam S.J., Kim S., Bai S.W., Chung B.C. (2009). Mass Spectrometry Based Metabolomic Approaches in Urinary Biomarker Study of Women’s Cancers. Clin. Chim. Acta.

[88] Guo M., Zhang L., Du Y., Du W., Liu D., Guo C., Pan Y., Tang D. (2018). Enrichment and Quantitative Determination of 5-(Hydroxymethyl)-2’-Deoxycytidine, 5-(Formyl)-2’-Deoxycytidine, and 5-(Carboxyl)-2’-Deoxycytidine in Human Urine of Breast Cancer Patients by Magnetic Hyper-Cross-Linked Microporous Polymers Based on Polyionic Liquid. Anal. Chem..

[89] Chen Y., Zhang R., Song Y., He J., Sun J., Bai J., An Z., Dong L., Zhan Q., Abliz Z. (2009). RRLC-MS/MS-Based Metabonomics Combined with in-Depth Analysis of Metabolic Correlation Network: Finding Potential Biomarkers for Breast Cancer. Analyst.

[90] Zahran F., Rashed R., Omran M., Darwish H., Belal A. (2021). Study on Urinary Candidate Metabolome for the Early Detection of Breast Cancer. Indian. J. Clin. Biochem..

[91] Silva C.L., Olival A., Perestrelo R., Silva P., Tomás H., Câmara J.S. (2019). Untargeted Urinary 1H NMR-Based Metabolomic Pattern as a Potential Platform in Breast Cancer Detection. Metabolites.

[92] Yu L., Jiang C., Huang S., Gong X., Wang S., Shen P. (2013). Analysis of Urinary Metabolites for Breast Cancer Patients Receiving Chemotherapy by CE-MS Coupled with on-Line Concentration. Clin. Biochem..

[93] Hu J.J., Takita C., Reis I.M., Yang G., Zhao W., Lee E. (2022). Abstract 2328: Metabolomics Pathways and Biomarkers in Predicting Breast Cancer Prognosis. Cancer Res..

